# α-ketoglutarate accumulation orchestrates immunosuppressive metabolic remodeling to drive cetuximab resistance in metastatic colorectal cancer

**DOI:** 10.1038/s41419-026-09001-8

**Published:** 2026-06-30

**Authors:** Yingwei Zhu, Kuijie Liu, Ling Chang, Jian Lu, Hong Tang, Zhenxin Zhu, Zhiru Chen, Xiongjun Wang, Jing Xiao

**Affiliations:** 1 Department of Gastroenterology, Jiangnan University Medical Center (JUMC), Wuxi, China; 2 Department of General Surgery, The Second Xiangya Hospital, Central South University, Changsha, China; 3 Department of Gastroenterology, The Seventh People’s Hospital of Shanghai University of Traditional Chinese Medicine, Shanghai, China; 4 Department of Pathology, Jiangnan University Medical Center (JUMC), Wuxi, China; 5 Gastrointestinal Surgery Department, Shanghai Changzheng Hospital, Second Military Medical University, Shanghai, China; 6 The Affiliated Guangdong Second Provincial General Hospital of Jinan University, Guangzhou, China; 7 Department of Clinical Laboratory Medicine, Shanghai Tenth People’s Hospital, School of Medicine, Tongji University, Shanghai, China; 8 School of Life Sciences, Guangzhou University, Guangzhou, China

**Keywords:** Cancer metabolism, Cancer microenvironment

## Abstract

Cetuximab resistance remains a major obstacle in the treatment of metastatic colorectal cancer (mCRC), highlighting the urgent need to identify synthetic lethal partners of EGFR. In this study, we observed glutamate dehydrogenase 1 (GDH1) accumulation in cetuximab-treated CRC samples. GDH1 depletion sensitized CRC cells to cetuximab and suppressed remodeling of the tumor immune microenvironment (TIME), as revealed by single-cell RNA sequencing. Mechanistically, cetuximab treatment induced substantial cytosolic accumulation of GDH1. Under normal conditions, EGFR directly phosphorylates cytosolic GDH1 at Y451, leading to HIP1R-mediated lysosomal degradation. Cetuximab, however, blocks GDH1-Y451 phosphorylation, thereby stabilizing GDH1 and increasing α-ketoglutarate (αKG) production. Elevated αKG enhances ALKBH5 activity to demethylate m6A modifications in the 3′UTR of *NDUFA2*, *CXCL3*, and *SOS1* pre-mRNAs. This cascade coordinately rewires tumor cell metabolism and reprograms the TIME, while also amplifying KRAS-driven signaling to promote CRC liver metastasis. Importantly, combining cetuximab with the GDH1 activity inhibitor R162 curbed tumor metabolic adaptation, reversed TIME remodeling, and suppressed KRAS activation, thereby preventing immune escape and metastatic progression. Our findings unveil the EGFR/GDH1/αKG/ALKBH5 axis as a key modulator of cetuximab response and suggest that post-treatment monitoring of blood αKG may help identify patients who could benefit from GDH1 inhibition to augment immunotherapy and KRAS-targeted strategies.

## Introduction

Cetuximab, a chimeric monoclonal antibody targeting epidermal growth factor receptor (EGFR), has been widely used in the treatment of colorectal cancer (CRC), especially at the stage of metastasis [1]. Cetuximab is also used in combination with chemotherapy or targeted agents in other cancers, such as bevacizumab for glioma [2] and paclitaxel for advanced breast cancer [3]. However, most patients with metastatic CRC (mCRC) develops cetuximab resistance which shortens the lifespan of mCRC cancer patients [4]. Thus, to recover the sensitivity of mCRC to cetuximab is imperative.

Tyrosine kinase receptor (RTK) signals control the activity of some metabolic enzymes and provide a new layer to target RTK signal. For example, EGFR constitutive activation and EGFR amplification drive serine metabolism and lactate synthesis [5]. In addition, EGFR directly phosphorylates Y148 of PKM2 and inhibits PKM2 enzyme activity [6]. Hence, it is reasonable to explore the correlation between metabolic remodeling and cetuximab drug resistance. Additionally, since some metabolic enzymes are closely related to RTK activity, the change of the related metabolites upon treatment of RTK inhibitors or antibodies may give a hint to indicate the disease progression. Glutamate dehydrogenase (GDH) is linked to glutamate metabolism and the tricarboxylic acid cycle through its metabolite αKG [7]. However, we do not fully understand whether GDH1 plays a role in cancer treatment.

N6-methyladenosine (m6A) is the most prevalent internal modification present in the messenger RNA (mRNA) across higher eukaryotes. It affects the stability and translation of modified transcripts, providing a mechanism for coordinated regulation of groups of transcripts mediated by writer, eraser, and reader proteins during cell fate transitions and tumour progression^(8)^. In this study, cytosolic GDH1 increased nuclear αKG drives ALKBH5 but not FTO to demethylate m6A in the 3’UTR of *NDUFA2, CXCL3* and *SOS1* pre-mRNA, then upregulates their stability. NDUFA2 is a subunit of mitochondrial complex 1, essential for oxidative phosphorylation (OXPHOS). For tumour cells, except energy balance, shaping an immune-microenvironment is of paramount importance. The upregulation of cytokines/chemokines, such as CXCL3, was proven to recruit more oncogenic neutrophils, macrophages, and other immune cells in tumours for TIME remodeling [8, 9]. More importantly, the accumulation of SOS1 would induce stronger activation of KRAS, especially mutant KRAS [10, 11].

In this study, our study revealed that EGFR-targeted therapy should be combined with GDH1 inhibitor, which disrupts the EGFR/GDH1/αKG/ALKBH5 signaling cascade and impairs tumour cell metabolic advantage, blocks the suitable TIME for tumour evasion and dampens KRAS activation. More importantly, following cetuximab treatment, the change of metabolite αKG may guide further immunotherapy and KRAS inhibition which prevents CRC-liver metastasis.

## Results

### GDH1 depletion sensitized CRC cells to cetuximab and blocked CRC cells-liver metastasis by reducing tumour-promoting immune-cells infiltration

**Fig. 1:**
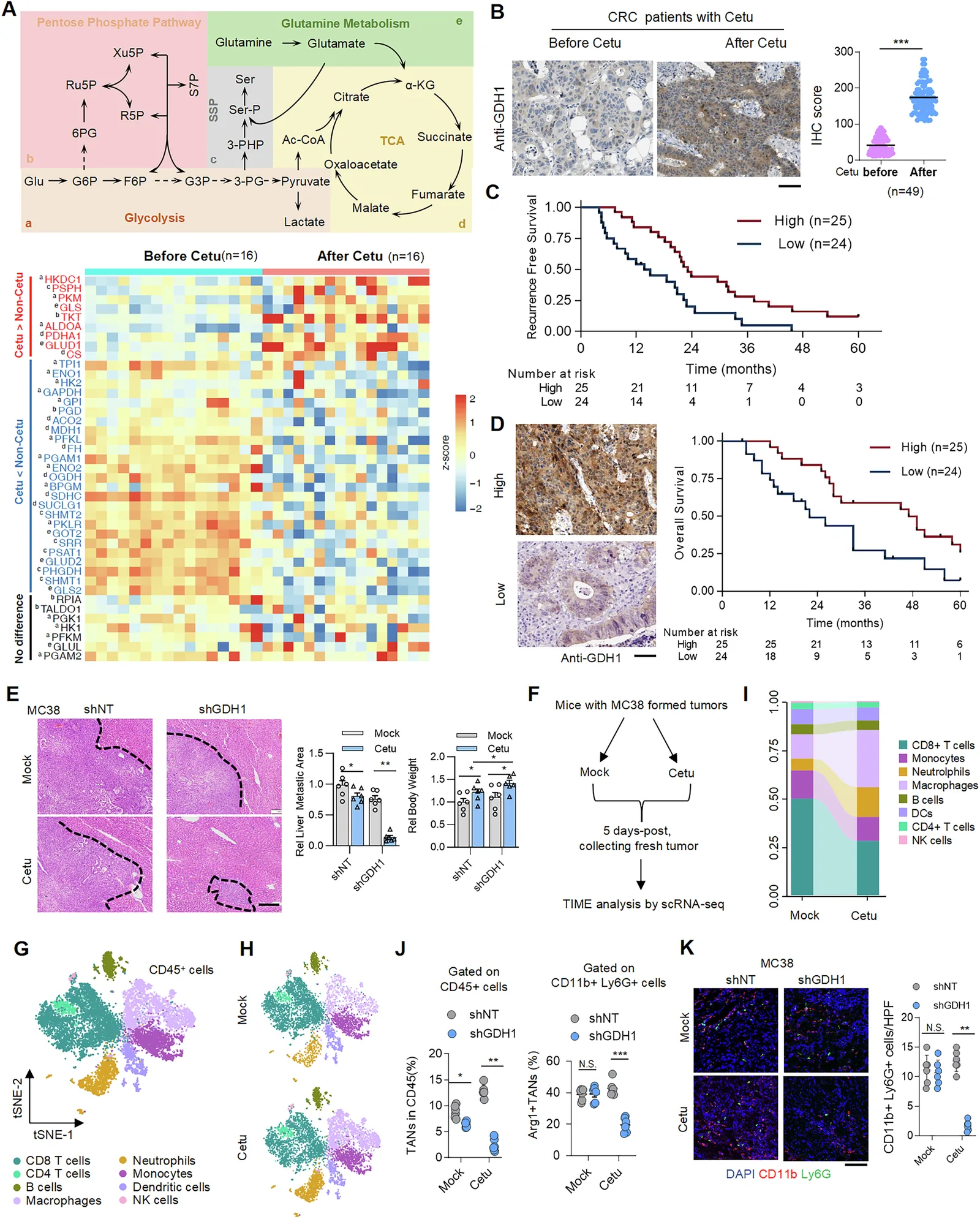
GDH1 is a risky factor for cetuximab treatment and its depletion blocked CRC cells-liver metastasis by reducing TANs infiltration. **A** The five metabolic pathways decomposing glucose and glutamine include glycolysis (a), pentose phosphate pathway (PPP, b), serine synthesis pathway (SSP, c), tricarboxylic acid cycle (TCA, d) and glutamine metabolic pathway (e) (Upper). CRC primary samples from patients receiving cetuximab (Cetu, *n* = 16) or not (*n* = 16) were collected for LC-MS/MS analysis and the protein intensities of important metabolic enzymes involving in above five pathways were extracted. The calculation result for heatmap was shown (Lower). **B** IHC quantification of 49 CRC samples receiving Cetuximab was performed using antibody against GDH1. Representative images are shown in (Left) while the IHC scores for GDH1 in above CRC samples was shown in (Right). Tumor samples were collected from 49 CRC patients after Cetuximab treatment and stained with antibody against GDH1. The recurrence free survival (RFS, **C**) and overall survival (OS, **D**) of these CRC patients were analyzed by a Kaplan‒Meier plot according to the group with GDH1 high or low intensity. Scale bar, 200 μm. **E** Adapted MC38 cells with or without GDH1 were injected into cecum. After Cetuximab, the livers were separated for H&E staining and the mice body weight was also measured. *n* = 6. Single-cell sequencing of MC38 formed tumors with or without Cetuximab treatment (**F**). UMAP plot depicting major cell types identified through unsupervised clustering analysis (**G**). Split UMAP visualization comparing cellular distributions across experimental conditions as indicated (**H**). Stacked bar chart quantifying the relative proportions of each cell type within MC38 formed tumors with or without Cetuximab treatment (**I**). *n* = 3. Flow cytometry (FCM) verified the percentage change of total TANs and Arg1^+^TANs in CD45^+^ immune cells (**J**); IF assay with antibodies against CD11b and Ly6G was performed to verify TANs in mice CRC tumors (**K**). Scale bar, 200 μm. *n* = 6 mice in each group. In vivo, mice were randomly assigned to treatment groups. (Data are presented as mean ± SEM. NS not significant; **p < 0.01; ***p < 0.001).

Glucose and glutamine serve as essential metabolic substrates for tumor cells, fueling bioenergetics, facilitating macromolecular biosynthesis, and sustaining rapid proliferation through metabolic reprogramming—a phenomenon epitomized by the Warburg effect. These pathways are thus regarded as critical targets for anticancer therapy. To delineate the interplay between glucose/glutamine catabolism and cetuximab response, we analyzed paired colorectal cancer (CRC) samples collected before and after cetuximab treatment, focusing on key enzymes involved in glycolysis, the pentose phosphate pathway (PPP), serine synthesis, the tricarboxylic acid (TCA) cycle, and glutamine metabolism. Our analysis revealed upregulation of nine metabolic enzymes following cetuximab exposure, among which glutamate dehydrogenase 1 (GDH1, also known as GLUD1) exhibited the most pronounced increase (Fig. 1A). GDH1 serves as a critical metabolic node interfacing the TCA cycle with glutamine catabolism via its product α-ketoglutarate (α-KG), thereby balancing flux between these two central pathways. Consistent with this role, GDH1 was significantly elevated across all 49 cetuximab-treated CRC specimens (Fig. 1B). Moreover, high GDH1 expression was identified as a prognostic risk factor associated with shortened recurrence-free survival and overall survival in patients with advanced CRC (Fig. 1C, D).

GDH1 depletion reduced the intracellular αKG level by 25% to 40%, proving αKG as a GDH1-generated product (Fig. S1A, B) and sensitized CRC cells to cetuximab in vitro (Fig. S1C). To truly mimic the colon-liver metastasis in advanced CRC patients, we trained MC38 parent cells to adapt to the liver microenvironment (Fig. S1D). Using liver-adapted MC38 cells, in vivo data demonstrate that GDH1 is required for colorectal cancer cells to withstand cetuximab (Fig. 1E). To explore sensitization strategies enhancing the anti-tumor efficacy of cetuximab, we further employed single-cell RNA sequencing (scRNA-seq) to characterize changes in the immune microenvironment following cetuximab treatment. In detail, scRNA-seq of MC38 cells formed orthotopic tumor tissues with or without GDH1 yielded 20416 high-quality cells by enriching CD45^-/+^ cells (Fig. S1E–G), revealing 8 distinct cell clusters (Fig. 1F, G). Analysis showed the increased proportions of neutrophils and macrophages in cetuximab-treated tumors compared to mock group, with neutrophils showing the most pronounced expansion while CD8^+^ T cell representation was markedly reduced (Figs. 1H, I and S1H, I). Furthermore, in primary tumours formed by GDH1-depleted MC38 cells, myeloid populations in the TIME, such as tumour-associated ARG1-positive neutrophils (Arg1^+^TANs) and CD206-positive macrophages (D206^+^TAMs) which are often ssociated with tumor-promoting/immunosuppressive functions, were decreased, while CD8^+^ T cells increased, showing GDH1-depleted tumor cells might sustain an anti-oncogenic immune-microenvironment (Figs. 1J, K and S1J). Similarly, clinical CRC samples showed high GDH1 positively correlated TANs recruitment (Fig. S1K). Combining cetuximab and GDH1 depletion prolonged the lifespan of mice receiving spleen-injected MC38 cells (Fig. S1L), indicating GDH1 should be a potential target to improve cetuximab treatment.

Therefore, we preliminarily explored the regulatory role of GDH1 in TIME and cancer treatment resistance.

### Cetuximab treatment induced the dephosphorylation of GDH1 at Y451, directing GDH1 accumulation in the cytosol, further reactivating ERK and AKT pathways

**Fig. 2:**
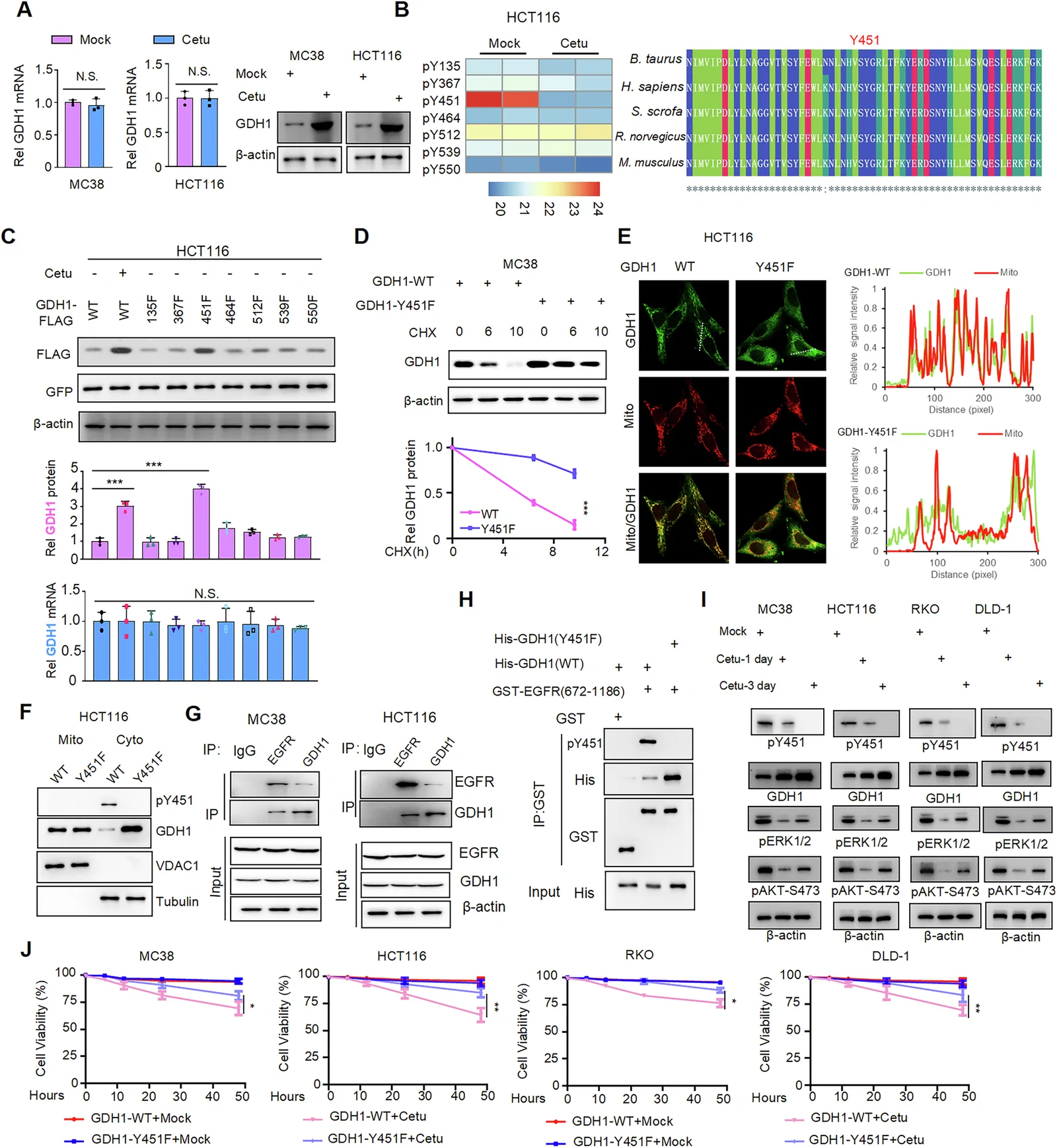
Cetuximab induces GDH1 accumulation in the cytosol which reactivates ERK and AKT pathway. **A** MC38 or HCT116 cells were treated with Cetu for 12 h and then collected for qRT‒PCR (A, Left) or WB (A, Right). *n* = 3. **B** HCT116 cells stably expressing GDH1-FLAG were treated with Cetu for 30 min, and GDH1-FLAG was enriched for a LC/MS analysis of tyrosine phosphorylation. Log2-transformed data was shown as a heatmap (Left). *n* = 2; The alignment of multiple mammals’ GDH1 C terminal (Right). **C** HCT116 cells expressing GDH1-FLAG (including WT or mutants) were treated with or without Cetu for 12 h. WB analysis and qPCR tested the levels of GDH1 protein and mRNA, respectively. Relative GDH1 protein and mRNA were analyzed. *n* = 3. **D** MC38 cells expressing GDH1-WT or -Y451F were treated with CHX (2 μM) for the indicated time, then WB analysis was performed using the indicated antibodies. *n* = 2. IF and cytosol-mitochondrial fractionation assay were performed using HCT116 cells expressing GDH1-WT or -Y451F. GDH1 localization was indicated by a GDH1 antibody and MitoTracker (**E**). Scale bars: 50 μm. The fractionation efficiency and GDH1 localization were determined by a WB analysis using antibodies against VDAC1, Tubulin and GDH1 (**F**). *n* = 3. **G** In MC38 or HCT116 cells, Co-IP and following WB was performed using the indicated antibodies. *n* = 3. **H** An in vitro kinase assay was performed by incubating *E. coli-*derived His-GDH1 (including WT or Y451F mutant) and GST-EGFR (672-1186 residues) together in the reaction buffer. WB analyzed the status of EGFR phosphorylating GDH1 using the indicated antibodies. *n* = 3. **I** MC38, HCT116, RKO or DLD-1 cells were treated with Cetu for 1 or 3 days, then collected for WB using antibodies against ERK1/2 or AKT1 phosphorylation. *n* = 3. **J** MC38, HCT116, RKO or DLD-1 cells expressing GDH1-WT or -Y451F were treated with or without Cetu for the indicated time points. Cell viability was assessed by trypan blue. *n* = 3. (Data are presented as mean ± SEM. NS not significant; **p < 0.01; ***p < 0.001).

Following Cetuximab treatment, we observed upregulation of GDH1 protein expression without a corresponding increase in its mRNA levels (Fig. 2A). LC–MS analysis further revealed a pronounced reduction in phosphorylation at GDH1-Y451—a phylogenetically conserved residue in mammals (Figs. 2B and S2A). To assess the impact of Y451 phosphorylation on GDH1 stability, we transiently expressed FLAG-tagged wild-type (WT) or mutant GDH1 constructs in MC38 cells. The GDH1-Y451F mutant exhibited enhanced protein stability compared to WT-GDH1 and other mutants, despite comparable mRNA expression (Fig. 2C). We additionally generated an antibody specifically recognizing phospho-Y451 of GDH1 (Fig. S2B). To investigate the functional relevance of GDH1-Y451 phosphorylation in cetuximab resistance, we introduced a GDH1-Y451F point mutation into HCT116 cells via CRISPR–Cas9-mediated genome editing, thereby mimicking a constitutive dephosphorylation state at this site (Fig. S2C). Cycloheximide (CHX) chase assays demonstrated that while GDH1-WT protein was largely degraded within 10 hours after inhibition of new protein synthesis, the GDH1-Y451F mutant retained more than half of its initial level (Figs. 2D and S2D).

Compared with GDH1-WT, GDH1-Y451F mutant showed a robust increase in the cytosol confirmed by IF (Figs. 2E and S2E) and fractionation assay (Figs. 2F and S2F). As Cetuximab impaired EGFR meanwhile hampered GDH1-Y451 phosphorylation, we suspect that EGFR could be associated with GDH1. A Co-immunoprecipitation (Co-IP) assay verified this hypothesis in both MC38 and HCT116 cells (Fig. 2G). GST pulldown assay in vitro showed the direct interaction between GDH1 and EGFR, and the phosphorylation of GDH1-Y451 by EGFR (Fig. 2H). Interestingly, we observed reactivation of canonical ERK1/2 and AKT phosphorylation, which indicated a reactivated pathway, was observed following 3 days of Cetuximab treatment (Fig. 2I and S2G), suggesting emergence of an adaptive mechanism for Cetuximab tolerance. Consistently, GDH1-Y451F mutant conferred more tolerance to cetuximab in CRC cells, regardless of KRAS or BRAF mutation status (Figs. 2J and S2H).

Hence, we identified GDH1 as a novel substrate of EGFR and cetuximab could block GDH1-Y451 phosphorylation which prevents GDH1 degradation in the cytosol and contributes to cetuximab resistance.

### HIP1R targeted Y451-phosphorylated GDH1 and induced cytosolic GDH1 degradation by lysosomes

**Fig. 3:**
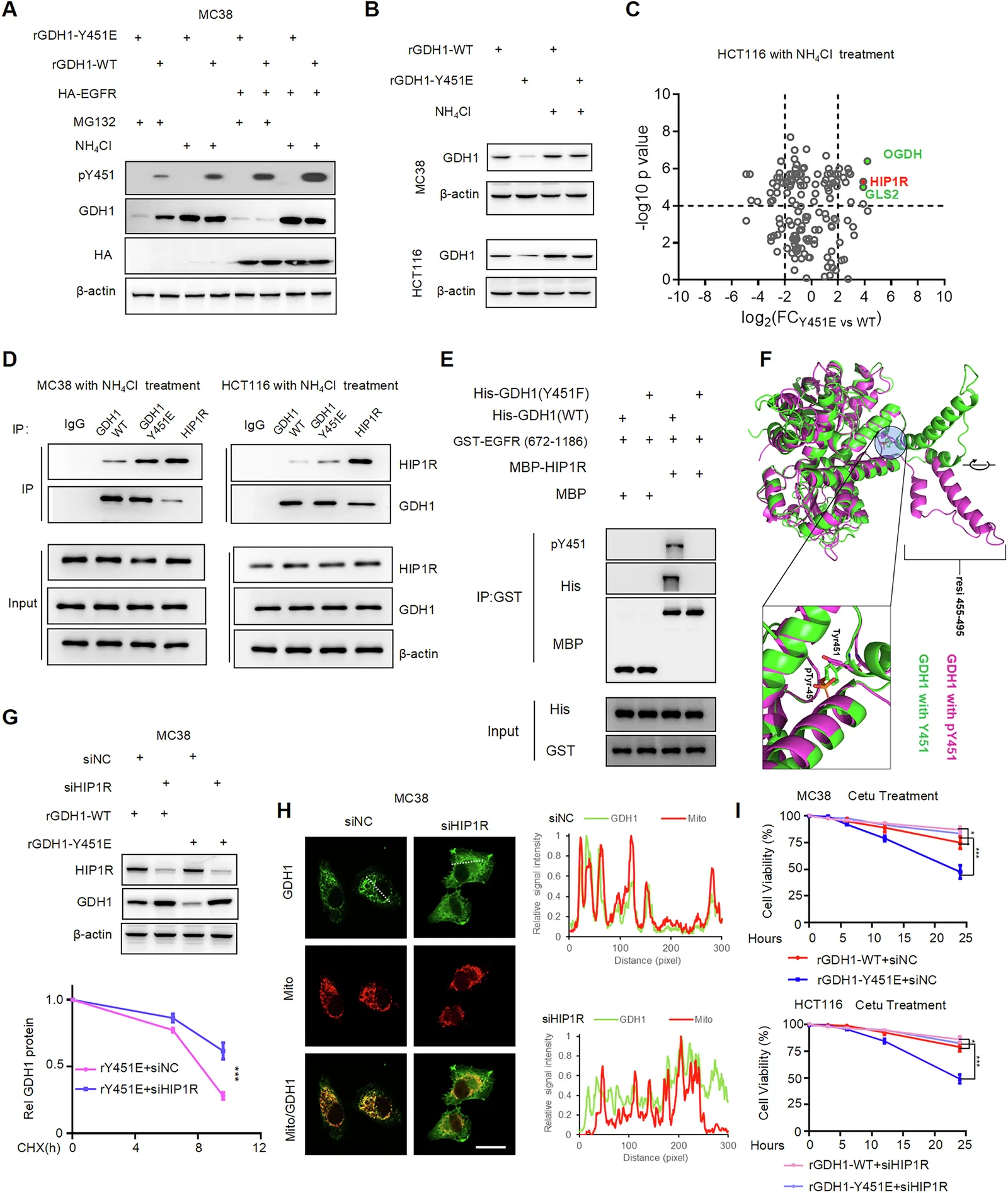
HIP1R targeted GDH1 with phosphorylated Y451 to support cytosolic GDH1 degradation by lysosomes. WB analysis of GDH1 protein stability. MC38 cells expressing GDH1-WT or -Y451E mutant was transfected with or without HA-EGFR. Then, MG132 or NH_4_Cl was added for 12 h (**A**). MC38 or HCT116 cells expressing GDH1-WT or -Y451E were treated with or without NH_4_Cl for 12 h (**B**). *n* = 3. **C** In HCT116 cells, FLAG-tagged GDH1 (WT or Y451E mutant) was transfected for 48 h, then the cells were treated with NH_4_Cl for 12 h. IP was performed using an antibody against FLAG, and the interactors of GDH1 (WT or Y451F mutant) were identified by LC‒MS and presented as a volcano plot. Red marks interesting candidate while Green marks the proteins identified as a GDH1-interactors with relatively high confidence. *n* = 3. **D** Lysates from cells in (**B**) were extracted for an endogenous Co-IP assay by separately incubating with an antibody against GDH1 or HIP1R. *n* = 3. **E** An in vitro kinase and GST-pulldown assay was performed by incubating *E. coli-*derived His-GDH1 (including WT or Y451F mutant), GST-EGFR (672-1186 residues) and MBP-tagged HIF1R together in the reaction buffer. WB analyzed the status of EGFR phosphorylating GDH1 and pulldown proteins using indicated antibodies. *n* = 3. **F** The phosphate is virtually placed at position Y451 of GDH1 by molecular dynamic simulation. **G** MC38 cells expressing GDH1-WT or -Y451E were transfected with siRNA targeting HIP1R (siHIP1R) mRNA for 48 h. The total proteins were extracted to test GDH1 and HIP1R (Upper). The protein quantity was calculated (Lower). *n* = 3. **H** MC38 cells were transfected with siHIP1R or siNC for 48 h. IF was performed to determine the subcellular location of GDH1 influenced by HIP1R depletion. *n* = 3. Scale bars: 50 μm. **I** siHIP1R or siNC was transfected into MC38 or HCT116 cells expressing GDH1-WT or -Y451E for 48 h and then treated with or without Cetu for the indicated times to assess cell viability. *n* = 3. (Data are presented as mean ± SEM. NS not significant; **p < 0.01; ***p < 0.001).

To elucidate the mechanism by which EGFR regulates GDH1 stability via Y451 phosphorylation, we first established stable cell lines expressing the phosphomimetic mutant GDH1-Y451E, which mimics constitutive phosphorylation at this residue without altering GDH1 mRNA expression (Fig. S3A, B). We then treated cells stably expressing GDH1-WT or GDH1-Y451E with either NH₄Cl, an inhibitor of lysosomal degradation, or MG132, a proteasome pathway inhibitor. Notably, NH₄Cl—but not MG132—effectively suppressed EGFR-mediated degradation of cytosolic GDH1 and restored protein levels reduced by the Y451E phosphomimetic mutation (Fig. 3A, B). These results demonstrate that EGFR regulates the stability of Y451-phosphorylated GDH1 primarily through the lysosomal degradation pathway. To identify proteins within this pathway that interact specifically with phospho-Y451 GDH1, we performed co-immunoprecipitation followed by mass spectrometry (IP–MS). This screen identified Huntingtin-interacting protein 1-related (HIP1R), OGDH, and GLS2 as specific binding partners of GDH1-Y451E (Fig. 3C).

HIP1R is a component of clathrin-coated pits and vesicles, may link the endocytic machinery to the actin cytoskeleton [12] and has been reported to be involved in PD-L1 degradation [13] while OGDH and GLS2 should utilize glutamate or α-Ketoglutarate as substrate and forms complex with GDH1 [14, 15]. The association of HIP1R and GDH1-WT or -Y451E was confirmed using forward and reverse Co-IP assays (Fig. 3D). Further, an in vitro GST pulldown assay showed negative mimic of Y451 phosphorylation abolished the interaction between GDH1 and HIP1R while EGFR phosphorylated GDH1 was captured by HIP1R (Fig. 3E).

We previously reported that cytosolic GDH1 was degraded by the RNF213-mediated ubiquitin degradation pathway [16]. However, co-immunoprecipitation assays revealed that GDH1-Y451E does not exhibit enhanced association with RNF213 compared to GDH1-WT (Fig. S3C). Structural modeling of the phosphate group at the GDH1-Y451 site indicated that the most favorable conformational change did not compromise GDH1 enzymatic activity. Instead, phosphorylation at Y451 may influence protein–protein interactions, as the phosphorylated form adopted a more open and flexible conformation (Figs. 3F and S3D). Depletion of HIP1R in cells expressing GDH1-Y451E restored GDH1 protein levels, underscoring the essential role of HIP1R in mediating the degradation of Y451-phosphorylated GDH1 (Figs. 3G and S3E). Notably, HIP1R knockdown led to the accumulation of cytosolic GDH1, while mitochondrial GDH1 levels remained unaffected (Figs. 3H and S3F). Consistent with these findings, loss of HIP1R largely restored cetuximab tolerance in GDH1-Y451E-expressing CRC cells (Fig. 3I). Taken together, these results demonstrate that HIP1R specifically regulates the abundance of cytosolic Y451-phosphorylated GDH1 by targeting it for lysosomal degradation.

### Cytosolic GDH1 increased nuclear αKG, which supported tumour immune evasion

**Fig. 4:**
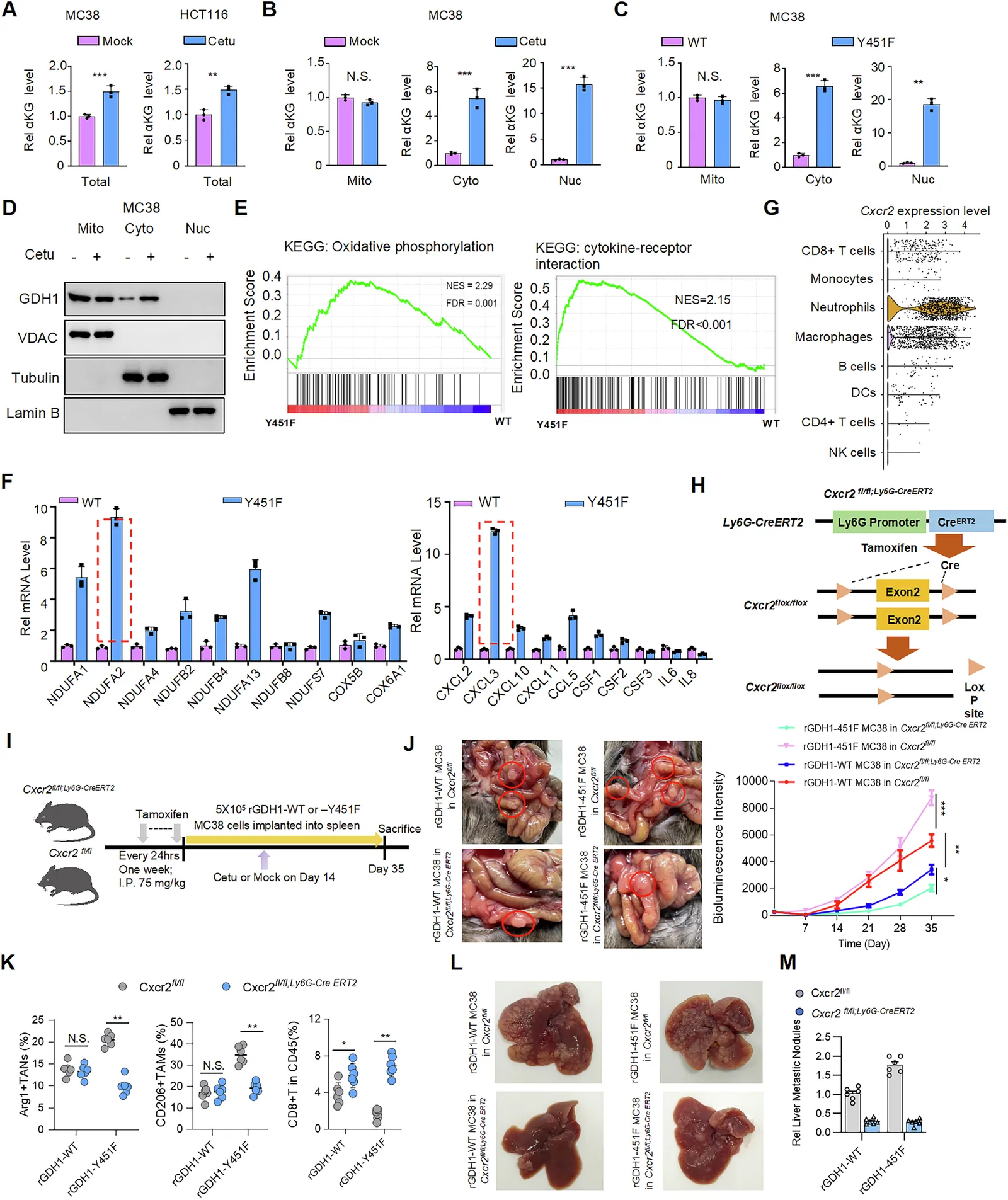
Cytosolic GDH1 directed a dramatic increase in nuclear αKG and induced gene expression of OXPHOS and chemokines. **A** MC38 or HCT116 cells were treated with or without Cetu for 12 h. αKG was measured by a kit and refers to the relative volume of collected cells. *n* = 3. Pretreated with or without Cetu for 12 h, the relative αKG levels in the cytosol, nuclei and mitochondria of MC38 wildtype cells (**B**) or MC38 cells expressing GDH1-WT or -Y451F (**C**) were sequentially fractioned and GDH1 distribution was ascertained by WB (**D**). Relative αKG was determined by comparing the volume versus αKG in the cytosol, nuclei and mitochondria. *n* = 3. RNA profiling was performed in HCT116 cells expressing WT- or Y451F-GDH1. GSEA analysis of genes ranking in GDH1-Y451F verse GDH1-WT for oxidative phosphorylation pathway or chemokine-receptor interaction pathway was shown (**E**). The expression of above two pathway involved genes was ascertained by qRT‒PCR (**F**). *n* = 3. **G** Violin plots illustrating *Cxcr2* expression levels across distinct cell populations in Fig. 1G. Genetic scheme for generating neutrophil-specific *Cxcr2* conditional knockout mice (*Cxcr2*^*fl/fl;Ly6g-CreERT2*^). Tamoxifen-inducible Cre recombinase under *Ly6g* promoter drives loxP-mediated excision of exon 2 in *Cxcr2* gene (**H**). Schematic diagram illustrating the orthotopic implantation model of MC38 cells in *Cxcr2*^*fl/fl*^ and *Cxcr2*^*fl/fl;Ly6g-CreERT2*^ mice. Mice received daily intraperitoneal (I.P.) injections of tamoxifen (75 mg/kg) for seven consecutive days prior to orthotopic implantation of 5 × 10^5^ rGDH1-WT or rGDH1-Y451F MC38 cells into the colorectal on Day 0. Cetuximab was administered on Day 14 post-implantation. Tumors were harvested on Day 35 for subsequent analyses, *n* = 6 (**I**). Bioluminescence intensity of MC38 cells formed tumors was measured and normalized to control mice group (**J**). Flow cytometry verified the percentage change of Arg1^+^TANs, CD206^+^TAMs, and CD8^+^ T cells in CD45^+^ immune cells within above tumors (**K**). The metastatic tumors in the livers were shown and calculated (**L, M**). In vivo mice were randomly assigned to treatment groups. (Data are presented as mean ± SEM. NS not significant; *p < 0.05; **p < 0.01; ***p < 0.001).

The mutation of Y451 to F451 had no significant effect on GDH1 enzymatic activity (Fig. S4A) while Cetuximab-treated cells induced an increase of total αKG by 50% (Fig. 4A), indicating that the observed phenotypes are driven by changes in GDH1 protein stability and subcellular localization rather than altered catalytic function. Moreover, Cetuximab treatment didn’t affect mitochondrial αKG but dramatically increased cytosolic and nuclear αKG (Fig. 4B). In line with this, a similar αKG distribution was observed in the GDH1-Y451F cells (Fig. 4C). Meanwhile, subcellular fractionation suggested a relative enrichment of αKG in the cytosol fraction upon cetuximab treatment (Fig. 4D). To detect the consequence of increased αKG, RNA-sequence was performed using the cells expressing restored GDH1-WT and -Y451F, and GSEA analysis revealed that OXPHOS and cytokine-receptor interaction pathways was enriched in the cells expressing GDH1-Y451F (Figs. S4B and 4E). We verified the TOP 10 genes in the above two pathways, and the most prominently upregulated genes in the Y451F mutant cells, i.e., *CXCL3* and *NUDFA2*, were selected as representative genes of the indicated pathways (Fig. 4F).

We generated stable cell lines expressing a nuclear-targeted variant of α-ketoglutarate dehydrogenase (OGDH) by fusing a C-terminal FLAG tag and nuclear localization signal (NLS), designated OFN (Fig. S4C, D). As shown, OFN expression effectively depleted nuclear α-ketoglutarate (α-KG) without significantly affecting mitochondrial or cytosolic α-KG pools (Fig. S4E). Depletion of nuclear α-KG led to downregulation of *NUDFA2* and *CXCL3* gene (Fig. S4F, G). Consistent with these observations, nuclear OGDH overexpression attenuated cellular tolerance to cetuximab in vitro (Fig. S4H). In parallel, treatment with the mitochondrial complex I inhibitors metformin or rotenone impaired cytosolic GDH1-dependent ATP generation (Fig. S4I), suggesting that α-KG produced by cytosolic GDH1 can enter mitochondria to support anaplerotic flux and enhance intracellular ATP production.

We analyzed CXCL3 expression in clinical CRC tumors with or without Cetuximab treatment and found that more CXCL3 was existed in tumors receiving Cetuximab (Fig. S4J). CXCR2 was the major receptor of CXCL3 [17] and expressed mainly in neutrophils and macrophages (Fig. 4G). We next employed a conditional knockout mouse model specifically targeting CXCR2 in neutrophils as neutrophils were are the foremost immune cells recruited into tumors. To circumvent developmental confounders, we crossed *Ly6g-CreERT2* inducible mice with *Cxcr2*-flox mice to generate *Cxcr2*^*fl/fl;Ly6g-CreERT2*^ mice, enabling tamoxifen-inducible ablation of CXCR2 expression specifically in neutrophils (Fig. 4H). PCR primers targeting the *flox* insertion site were designed to differentiate between the recombinant and intact alleles. PCR analysis confirmed effective Cre-mediated recombination at the loxP sites within the Cxcr2 locus following tamoxifen administration in *Cxcr2*^*fl/fl;Ly6g-CreERT2*^ mice, compared to control *Cxcr2*^*fl/fl*^ mice while CXCR2 protein in neutrophils by western blot analysis was clearly diminished (Fig. S4K, L).

Subsequently, for orthotopic implantation, *Cxcr2*^*fl/fl*^ and *Cxcr2*^*fl/fl;Ly6g-CreERT2*^ mice were intraperitoneally administered tamoxifen daily (75 mg/kg) for 7 consecutive days prior to orthotopic implantation of MC38 cells expressing rGDH1-WT or rGDH1-Y451F. On Day 14 post-implantation, cetuximab was administered. The orthotopic tumor tissues were monitored and calculated, then harvested on Day 35 for immune cells analysis (Fig. 4I). rGDH1-Y451F cells formed larger tumors but diminished upon Cxcr2 depletion in neutrophils (Fig. 4J). In TIME, Cxcr2 depletion in neutrophils reduced the populations of Arg1^+^TANs and CD206^+^TAMs while boosted CD8^+^ T infiltration in tumors (Fig. 4K), consistently, dampened the liver metastasis of MC38 cells regardless of tumor cells with or without GDH1-Y451F mutation (Fig. 4L, M).

Taken together, dephosphorylation at Y451 stabilized cytosolic GDH1, leading to robustly increased nuclear αKG, then conferring tolerance to cetuximab, probably by upregulating *NUDFA2* and *CXCL3* which coordinately drive OXPHOS and TIME remodeling.

### Cytosolic GDH1 mediated the increased nuclear αKG activates ALKBH5, epigenetically stabilizing *NUDFA2, CXCL3* and *SOS1* mRNA

**Fig. 5:**
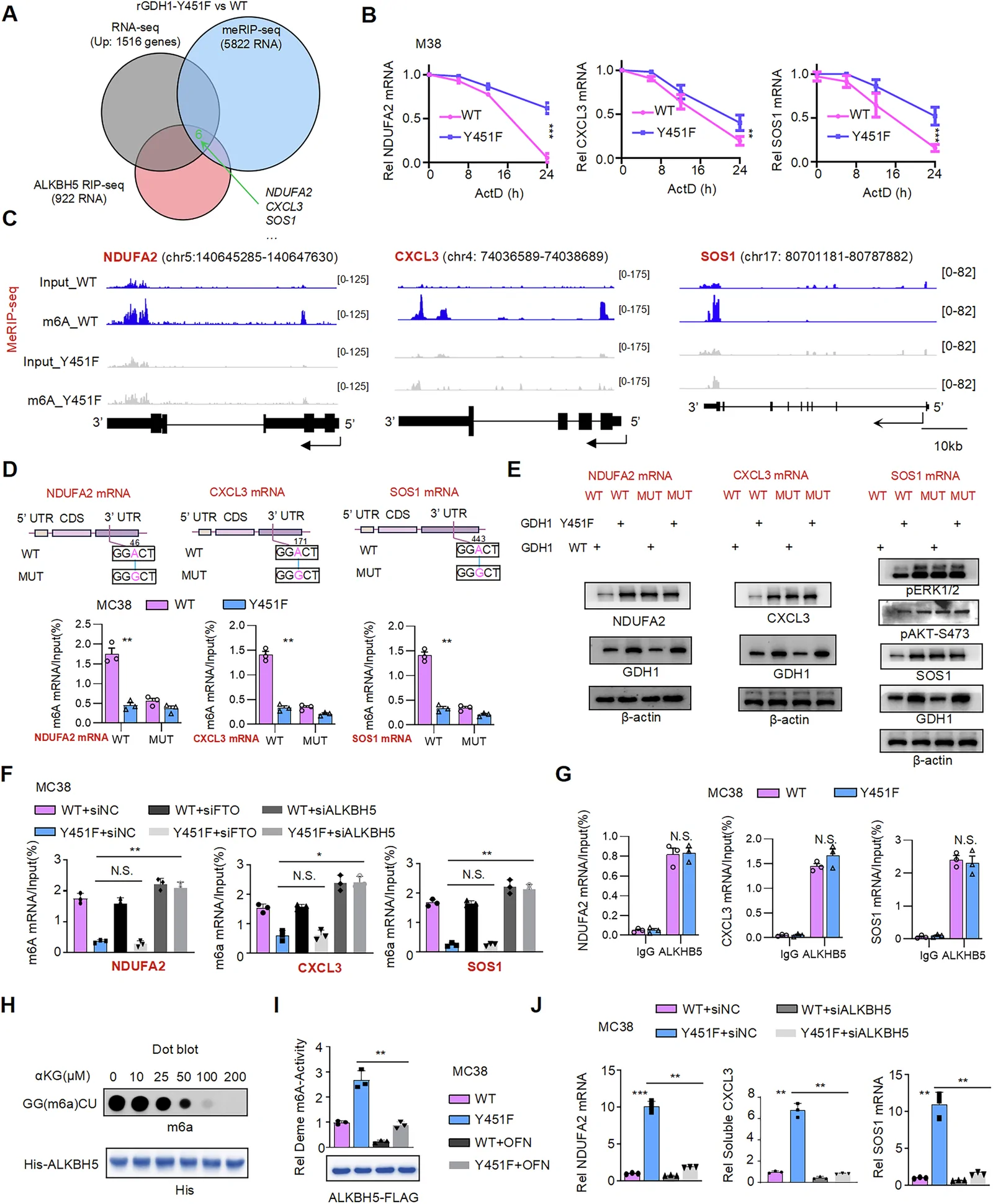
Cytosolic GDH1 regulates *NDUFA2/CXCL3/SOS1* mRNA stability by triggering ALKHB5 activation. **A** The overlapped gene list of RNA-seq, methylated RNA IP (meRIP)-Seq and ALKBH5 RIP-Seq in MC38 cells expressing GDH1-WT or -Y451F mutant, includes *NUDFA2*, *CXCL3* and *SOS1*. **B** In MC38 cells expressing GDH1-WT or -Y451F mutant, mRNA decay assay was performed by measuring mRNA level using qRT-PCR in the indicated time points. *n* = 3. MeRIP-seq was performed using HCT116 cells expressing GDH1-WT or -Y451F mutant. Normalized m6A read density levels of *NUDFA2*, *CXCL3* and *SOS1* mRNA are shown (**C**). m6A levels of *NUDFA2*, *CXCL3* and *SOS1* mRNA in indicated MC38 cells with or without adenosine mutation were identified by MeRIP-qPCR using anti-IgG and anti-m6a antibodies (*n* = 3, **D**). WB analysis of NUDFA2, CXCL3 and SOS1 proteins in above cells was shown (**E**). *n* = 3. **F** After FTO or ALKHB5 depletion, m6A levels of *NUDFA2*, *CXCL3* and *SOS1* mRNA were identified (*n* = 3). **G** In MC38 cells expressing WT- or GDH1-Y451F, RIP-qPCR validated that the target of ALKBH5 to *NUDFA2*, *CXCL3* and *SOS1* mRNA. Relative enrichment of *NUDFA2*, *CXCL3* and *SOS1* mRNA in each group was shown with the normalization to input. *n* = 3. **H** Dot blotting was performed to test ALKHB5 activity, by incubating His-ALKHB5 with m6a-modified GG(m6a)CU sequence RNA. Antibody against m6a was used to measure ALKHB5 activity under different dose αKG from 0 to 200 μM. *n* = 3. **I** ALKHB5 activity test using HCT116 expressed FLAG-ALKBH5. *n* = 3. **J** In MC38 or HCT116 cells expressing GDH1-WT or -Y451F mutant with or without ALKBH5 depletion, *NUDFA2*, *CXCL3* and *SOS1* mRNA were measured using qRT-PCR. *n* = 3. (Data are presented as mean ± SEM. NS not significant; *p < 0.05; **p < 0.01; ***p < 0.001).

RNA abundance is tightly regulated by transcriptional and post-transcriptional level [18]. In this study, given that elevated nuclear αKG potentiates the enzymatic activity of dioxygenases such as the mRNA m⁶A demethylases ALKBH5 and FTO [19], we first explored the effects of the GDH1-Y451 phosphomimic mutation on post-transcriptional regulation. To identify candidate targets, we performed methylated RNA IP (meRIP)-Seq and ALKBH5 RIP-Seq and observed the overlapped mRNAs including *NUDFA2*, *CXCL3* and *SOS1* (Fig. 5A). To test if these mRNAs were stabilized, we performed RNA decay assays and found, in the cells expressing GDH1-Y451F, *NUDFA2*, *CXCL3* and *SOS1* mRNA were more stabilized (Figs. 5B and S5A). As m6A modification plays a critical role in mRNA stability [20], we next asked if it was involved. meRIP-sequencing examined m6A levels in CRC cells expressing GDH1-WT or -Y451F. As shown, 3’UTR of *NUDFA2*, *CXCL3* and *SOS1* pre-mRNAs was modified by m6A which was significantly impaired in the cells expressing GDH1-Y451F (Fig. 5C). To establish a direct link, we mutated the potential adenosine sites which should be methylated and performed RIP-qPCR. As shown, compared to WT group, mRNA adenosine methylation was decreased in the adenosine mutated groups (Fig. 5D). Consequently, NUDFA2, CXCL3 and SOS1 proteins were upregulated in the adenosine to guanosine mutated groups (Fig. 5E).

At present, both FTO and ALKBH5 were two major dioxygenases required for demethylating m6A-modified mRNA [21]. To determine the responsible demethylase, we depleted FTO or ALKBH5. Only ALKBH5 depletion recovered m6A levels on the target mRNAs (Fig. 5F). Since ALKBH5 is a nuclear protein, its activation is likely most sensitive to the local nuclear αKG concentration. Our observation that nuclear-targeted OGDH (OFN) depletes nuclear αKG (Fig. S4E) supports the functional importance of the nuclear αKG pool in this context. We found that ALKBH5 bound these mRNAs, but its enrichment did not increase in GDH1-Y451F cells (Figs. 5G and S5B), suggesting cytosolic GDH1 increased nuclear αKG, upregulating ALKBH5 activity but not the enrichment on target mRNA.

Accompanied by the increased αKG, ALKBH5 utilizes αKG as a cofactor to drive its own activation [22]. A dot blotting assay showed efficient activation at 50–100 μM αKG (Fig. 5H), and ALKBH5 from GDH1-Y451F cells had upregulated activity (Fig. 5I). Functionally, Loss of ALKBH5 diminished the increased target gene expression, upregulated by GDH1-Y451F (Figs. 5J and S5C). In the cells expressing GDH1-Y451F, the soluble CXCL3 was also recoverably reduced after ALKDH5 depletion (Fig. S5C). Western blotting analysis showed a consistent result with *NUDFA2* and *SOS1* mRNA (Fig. S5D). Consequently, ALKBH5 abolished the robust ATP production induced by ALKBH5 increased NDUFA2 (Fig. S5E) and stronger KRAS activity induced by ALKBH5 upregulated SOS1 (Fig. S5F, G). In vitro survival assays showed ALKDH5 depletion sensitized colorectal cancer cells to cetuximab (Fig. S5H).

Using TCGA database with mRNA levels in CRC clinical samples, ALKBH5 showed a positive correlation with the *NUDFA2*, *CXCL3* and *SOS1* mRNA levels, further proving ALKBH5 should stabilize above mRNAs (Fig. S5I).

Taken together, these data demonstrated that EGFR/GDH1/αKG/ALKBH5 axis mediated the stability of *NUDFA2*, *CXCL3* and *SOS1* mRNA, then produced excessive ATP and KRAS activity.

### The combination of cetuximab plus R162 efficiently restrained CRC progression by blocking TIME remodeling

To suppress the elevated nuclear α-ketoglutarate (α-KG) resulting from cytosolic GDH1 activity, we administered the GDH1 activity inhibitor R162 at both low and high doses. While high-dose R162 (H-R162) effectively suppressed tumor growth, it induced notable liver toxicity (Fig. S6A, B). Therefore, a low-dose R162 (L-R162) was selected for combination treatment with cetuximab in CRC cells. This combination regimen resulted in a synergistic suppression of CRC growth in vivo (Fig. S6C). Cellular fractionation analysis confirmed that L-R162 reduced α-KG levels in both the cytosol and nucleus to below 15 μM, a concentration insufficient for effective activation of the demethylase ALKBH5 (Fig. S6D).

**Fig. 6:**
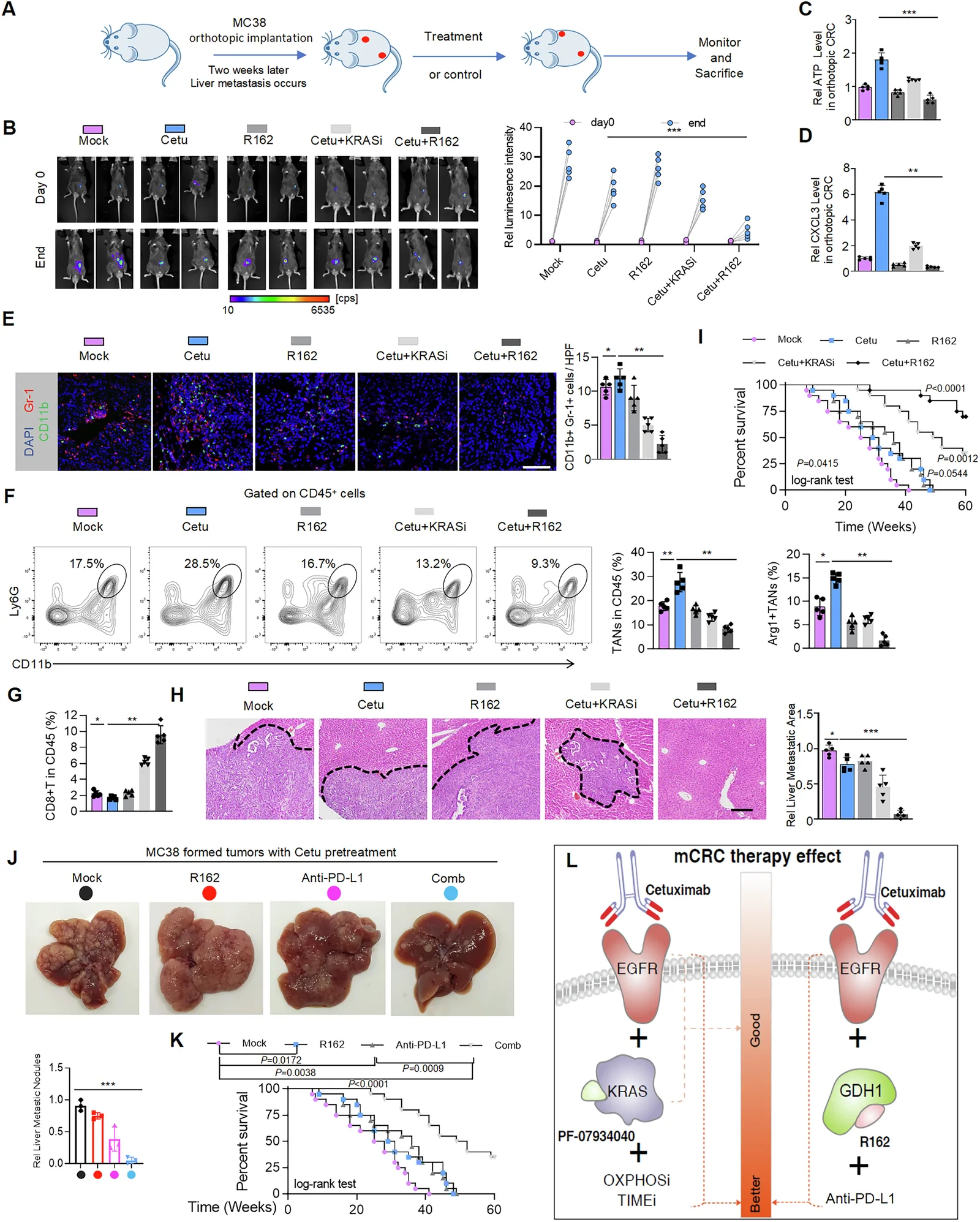
The combination of cetuximab plus R162 efficiently restrained CRC progression by blocking TIME remodeling. Experimental design for measuring orthotopic CRC TIME and CRC-liver metastasis using the combination of cetuximab with KRAS inhibitor PF-07934040 or GDH1 inhibitor R162. MC38 cells were orthotopically implanted into the cecum wall of C57BL/6 mice and executed with the indicated treatment (**A**). The in vivo growth of orthotopic MC38 cells was monitored by bioluminescence imaging (**B**). The quantity of intracellular ATP and soluble CXCL3 in orthotopic MC38 formed tumors were also analyzed (**C, D**). The percentages of tumor associated granulocytes (**E**), total neutrophils and Arg1+ neutrophils (**F**), and CD8^+^ T (**G**) in CD45^+^ cells were assessed using IF or FCM. *n* = 5 mice in each group. Liver metastasis was confirmed using H&E (**H**). Scale bar, 500 μm. *n* = 5 mice in each group. The overall survival of mice under the indicated treatment conditions was analyzed by a Kaplan‒Meier plot (**I**). *n* = 20 mice in each group. MC38 formed tumors were pretreated with Cetu until the resistance occurred. Then, L-R162 or anti-PD-L1 antibody alone or combination was executed. The livers were separated and shown (**J**, upper) while the metastatic nodules in livers were calculated (**J**, down). The overall survival of mice under the indicated treatment conditions was analyzed by a Kaplan‒Meier plot (**K**). *n* = 20 mice in each group. In vivo mice were randomly assigned to treatment groups. **L** The advantages of combining Cetu and R162 for treating mCRC. (Data are presented as mean ± SEM. **p < 0.01; ***p < 0.001).

To assess TIME remodeling and liver metastasis under combination therapy, we established an orthotopic CRC model by implanting MC38 cells into the cecal wall of C57BL/6 mice, followed by treatment with cetuximab and R162 (Fig. 6A). The combination of cetuximab and R162 significantly inhibited orthotopic tumor growth more effectively than either agent alone, and outperformed the combination of cetuximab with the KRAS inhibitor PF-07934040 (Figs. 6B and S6E). Consistent with these findings, protein levels of NDUFA2, CXCL3, and SOS1 were downregulated upon R162 treatment (Fig. S6F, G). Furthermore, ATP quantification and ELISA confirmed that R162 abrogated cetuximab-induced upregulation of intracellular ATP and CXCL3 in CRC tumors (Fig. 6C, D).

Chemokines, such as CXCL3, is secreted by tumour cells or immune cells to recruit TANs and TAMs, and the change of immune-cell interaction network is named as TIME remodeling [23, 24]. Immunofluorescence staining confirmed decreased granulocyte infiltration in MC38 cells formed tumours with R162 treatment alone or combining cetuximab (Fig. 6E). In line with granulocyte infiltration, flow cytometry showed similar but more striking patterns of Arg1^+^TANs infiltration in MC38 cells formed tumours (Fig. 6F). Accordingly, as TAMs are recruited after neutrophils colonize the tumour area [25], the enrichment of TAMs in MC38 cells formed tumours was also downregulated (Fig. S6H). Consequently, the enrichment of CD8^+^ T cells was increased upon the indicated strategies (Fig. 6G).

H&E staining confirmed that orthotopic MC38 tumors derived hepatic metastasis, an effect that was substantially suppressed by the combination of cetuximab and L-R162. This regimen nearly eliminated metastatic liver lesions (Fig. 6H), increased mouse body weight (Fig. S6I), and significantly extended the survival of experimental animals (Fig. 6I). Consistently, a similar suppression trend was observed in intratumoral ATP levels, soluble CXCL3, and KRAS activity in MC38-derived liver metastases (Fig. S6J–L).

To further evaluate whether GDH1 inhibition can reverse established cetuximab resistance, we pre-treated orthotopic MC38 tumors with cetuximab until resistance developed, followed by administration of R162. In cetuximab-resistant colorectal cancer models, the combination of L-R162 with anti–PD-L1 antibody resulted in pronounced antitumor efficacy, particularly in suppressing liver metastasis and extending survival (Fig. 6J, K).

Collectively, these in vivo findings demonstrate that pharmacological inhibition of GDH1 enzymatic activity not only restricts tumor bioenergetics but also disrupts the ability of tumors to remodel the immune microenvironment. This dual mechanism underpins the superior therapeutic benefit of combining R162 with EGFR blockade, outperforming KRAS inhibitor–based regimens (Fig. 6L).

### The EGFR/GDH1 axis showed potential clinical significance in cetuximab treatment and αKG may guide immunotherapy

**Fig. 7:**
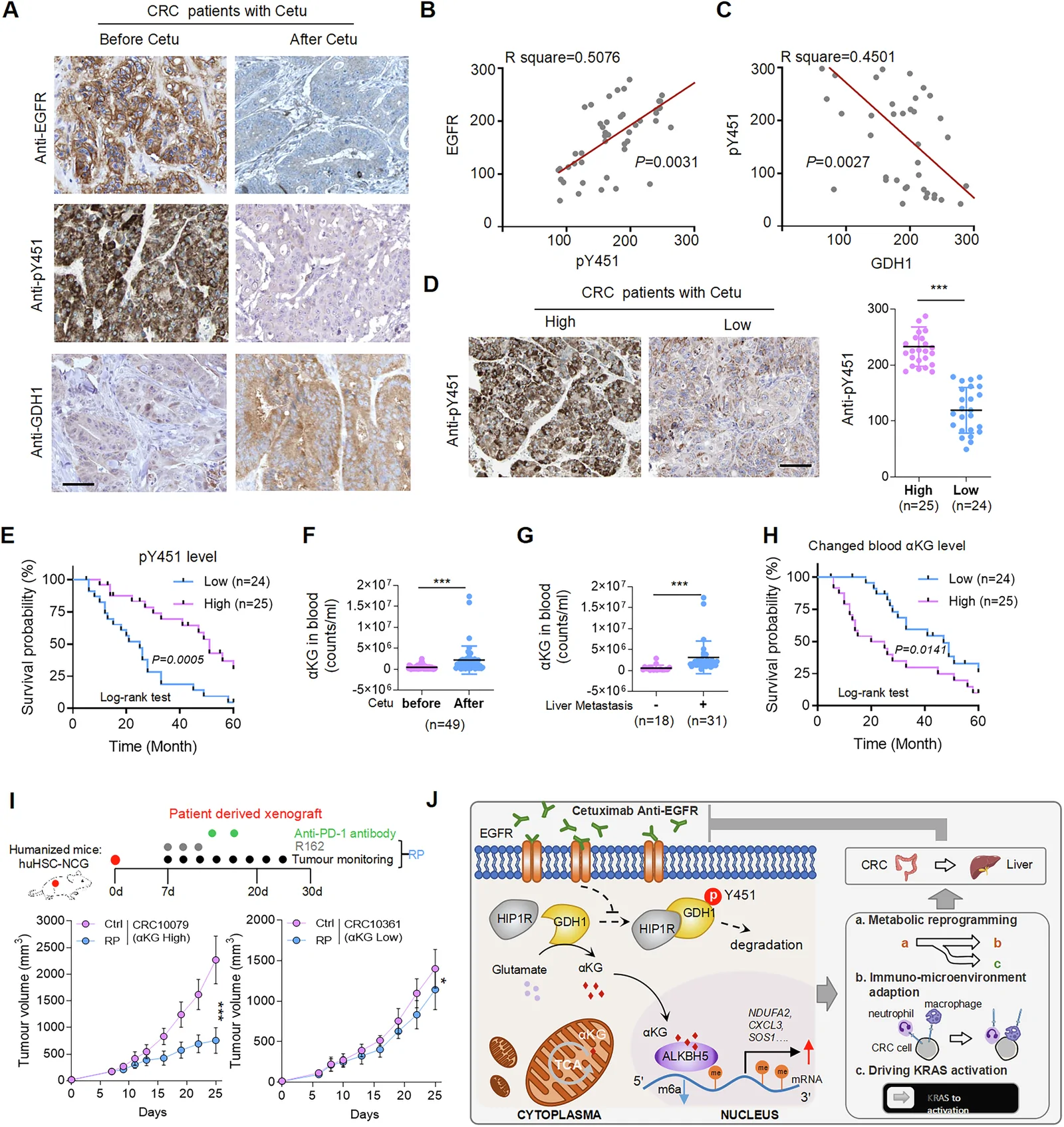
The EGFR/GDH1/HIP1R axis showed potential clinical significance and αKG the immunotherapy following cetuximab in liver-metastatic CRC. IHC quantification of above CRC samples was performed using antibodies against EGFR, pY451 or GDH1. Representative images are shown in (**A**). The correlated IHC signal between EGFR and pY451 (**B**) or pY451 and GDH1 (**C**) was calculated by personal correlation test. Scale bars: 200 μm. Clinical significance of pY451 in CRC progression. The median positive numbers were set as the cut-offs to divide the patients. Representative images of high and low expression of pY451 are shown in (**D**, Left), and the IHC score was calculated and analyzed as shown in (**D**, Right). The overall survival of the CRC patients indicated as pY451(**E**) was analyzed by a Kaplan‒Meier plot. Scale bars: 200 μm. Clinical significance of αKG in CRC progression. Blood from above CRC patients receiving cetuximab was retained and blood αKG was quantified using LC-MS (**F**). Blood αKG from CRC patients with or without liver metastasis was calculated (**G**). The overall survival plot of the CRC patients who were divided by the median value of αKG change using the counts of αKG in “after group” minus that in “before group” (**H**). **I** Patient derived tumor bearing mice received RP treatment: R162 (GDH1 inhibitor) with anti-PD-1 antibody. Tumor volume was monitored after treatment. The vertical gray dotted line indicates R162 treatment and the green dotted line means the duration of PD-1 antibody administration. *n* = 6 mice in each group. In vivo mice were randomly assigned to treatment groups. **J** Schematic model of EGFR/GDH1/HIP1R axis-mediated αKG homeostasis controlling cetuximab resistance and dampening immunotherapy. (Data are presented as mean ± SEM. ***p < 0.001).

To test whether cetuximab treatment induces GDH1 stabilization in the clinic, we collected samples from 49 CRC patients receiving cetuximab. All antibodies used in IHC were ascertained by the indicated peptide or protein block assay (Fig. S7A). IHC staining showed that after cetuximab treatment, GDH1 protein was increased in the CRC tissues, while Y451 phosphorylation almost disappeared (Fig. 7A). Using the IHC score from the CRC samples before and after cetuximab treatment, we calculated above IHC intensities and ascertained the significant correlation between EGFR and GDH1-Y451 phosphorylation (Fig. 7B) or GDH1-Y451 phosphorylation and GDH1 protein (Fig. 7C), supporting the true existence of EGFR/GDH1 axis in the clinic.

We also tested GDH1-Y451 phosphorylation and HIP1R in these CRC samples and the IHC intensity score with the high or low levels of GDH1-Y451 phosphorylation and HIP1R was shown (Figs. 7D and S7B), and used to separate CRC patients into high or low two groups. The overall survival of these patients showed that higher expression of GDH1-Y451 phosphorylation or HIP1R was associated with better prognosis (Fig. 7E and S7C).

Before and after receiving treatment, the changes of metabolites in blood may be used as a noninvasive biomarker to monitor the effect of treatment, especially the occurrence of drug resistance. Hence, we also collected the CRC patients’ blood and tested the metabolite αKG using LC-MS. As shown, after cetuximab treatment, blood αKG increased (Fig. 7F), especially αKG from CRC patients with liver metastasis (Fig. 7G), implying αKG should be utilized as an indicator to predict CRC-liver metastasis. In line with the occurrence of liver metastasis, the overall survival of these patients showed that a higher level of blood αKG was associated with a worse prognosis (Fig. 7H). After cetuximab, to explore the potential efficacy of R162 and PD-1 blockade combined therapy (named RP treatment), the PDX model was utilized as follows: we inoculated tumour pieces derived from CRC patients with cetuximab treatment into cecum to 8-week-old sex matched humanized mice and treated them with or without RP. PDX with high level of αKG showed more effective response to RP treatment than the group with low level of αKG, indicating that patients harboring high level of αKG are more suitable for RP therapy (Fig. 7I).

Taken together, the data above proved the clinical relevance of EGFR/GDH1/HIP1R-mediated αKG axis in metastatic CRC patients. Additionally, after cetuximab treatment, αKG level in blood may guide the further treatment strategy by combining immunotherapy with R162.

## Discussion

In this study, we reveal that cetuximab treatment induces the dephosphorylation of GDH1 and prevents HIP1R from GDH1. Cytosolic GDH1 stabilization increases, and in turn, nuclear αKG is robustly enhanced. ALKBH5 mediated by comparatively high nuclear αKG, presented RNA demethylase activity on the 3’UTR of *NUDFA2*, *CXCL3* and *SOS1* pre-mRNA to endow CRC cells with metabolic advantages and immune-evasion. Blockade of cytosolic GDH1 impairs tumour cell survival following EGFR inhibition and dampens their immune-evasion which potentiates PD-1 immunotherapy (Fig. 7J). To clearly state our viewpoints, we summarize the significances and limitations of this study.

EGFR has a broad range of substrates which not only offer a proliferative advantage but also support tumour cell survival under disadvantageous stress [26], hence, targeting EGFR should be an effective strategy against pan-cancer. However, as EGFR is so important for tumour cells, blockade of EGFR by cetuximab would induce inevitable resistance. In this study, we confirmed that GDH1 is a novel substrate of EGFR and confers cetuximab adaptive tolerance.

EGFR-mediated phosphorylation of GDH1 was targeted by HIP1R which functions as a negative regulator of PD-L1 by targeting PD-L1 for lysosomal degradation [13]. In this study, we found that HIP1R directed cytosolic GDH1 to be degraded by the lysosomal pathway. However, our previous study discovered that the GDH1 protein is degraded via the ubiquitin–proteasome system [16]. Thus, we suspected that the precise GDH1 protein level controlled by different degradation pathways is determined by the cell context and stressful environment. The level of important proteins required for cell survival or growth is tightly controlled. For example, c-Myc is a driver of cell growth and metabolism, and multiple cellular controls act to regulate its levels. An important mechanism controlling the c-Myc levels is differential degradation via the ubiquitin–proteasome system [27] or lysosomal pathway [28].

We also proposed that the accumulation of cytosolic GDH1 was tightly related to the adverse environment of tumour cells. When tumour cells grow, both enough energy and mass are needed. At this stage, the TCA cycle is the major energy source, while glycolysis, amino acids and fatty acids supply the metabolites to build mass. GDH1, as a metabolic enzyme, links glutamate metabolism to TCA and integrates energy metabolism and mass building [29]. To support the growth of tumour cells, GDH1 is locked in the mitochondria and produces αKG to fuel the TCA cycle. However, when tumour cells encounter stresses, they would adjust their metabolic strategies to support survival. Hence, EGFR blockade by cetuximab increased cytosolic GDH1, which should be regarded as a backup survival strategy for tumour cells upon EGFR inhibition.

Compared with mitochondrial GDH1-produced αKG, cytosolic GDH1-produced αKG could be more conveniently dispersed into nuclei, to irritably modulate gene expression. The distribution of metabolites always depends on the subcellular localization of relevant enzymes. For example, in mammalian cells, acetyl-CoA is produced in mitochondria, the cytosol, and nuclei depending on the localization of related metabolic enzymes, such as a nuclear pyruvate dehydrogenase complex that exists in the nuclei and generates nuclear acetyl-CoA for histone acetylation [30]. To ascertain the robust increase of nuclear αKG in the cells expressing cytosolic GDH1, we constructed cells stably expressing nuclear OGDH which exhausted nuclear αKG and diminished the phenotype induced by increased cytosolic GDH1, further proving that the tolerance to cetuximab is due to an increase in nuclear αKG.

Furthermore, αKG levels are maintained by multiple pathways, such as glutaminolysis (via GLS), IDH1/2-mediated reductive carboxylation, and transaminase reactions. Our data showed no consistent upregulation of GLS or IDH1/2 after cetuximab treatment, suggesting that stabilized cytosolic GDH1 may be a dominant mechanism for increasing nuclear αKG to activate ALKBH5 in this context. The relative contributions of these parallel pathways warrant future study.The epigenetic role of αKG is well known as αKG functions as a cofactor of dioxygenases and plays a shadowy role in cancer [19]. Our previous study proved that KDM4A/6 A was activated once αKG reached 50 μM [16]. In this study, after EGFR blockade by cetuximab, nuclear αKG increased to 75 μM, which robustly activates ALKBH5, an RNA demethylase targeting NDUFA2 and CXCL3 mRNA for removing m6A modification.

Noninvasive testing has the huge advantage in monitoring tumour progression, however, there are still lack of the precise indicators for noninvasive testing. With the development of mass spectrometry, more metabolites are accurately quantified in blood. For tumour patients, changes in metabolites may guide follow-up treatment strategies. We measured blood αKG upon cetuximab treatment in CRC patients and mCRC patients have more abundant αKG, thus, blood αKG as an indicator for noninvasive testing may guide further treatment.

Through RNA profiling, meRIP-Seq and ALKBH5 RIP-Seq, we observed that stabilized GDH1 (Y451F mutant) robustly induced the expression of genes involved in OXPHOS, cytokine-chemokine and SOS1 mRNA abundance. Multiple studies have revealed that OXPHOS partially drives chemoresistance in cancer [31]. The inhibition of OXPHOS suppresses resistance to EGFR-RTK inhibitors in EGFR mutant-driven lung adenocarcinoma. Thus, OXPHOS is an emerging target in cancer therapy, and OXPHOS inhibitors, such as metformin or arsenic trioxide, has been demonstrated to be potential anticancer drugs [32]. Except for metabolic advantage, cytosolic GDH1 also supports tumour evasion. CRC cells accumulated cytosolic GDH1 also highly expressed chemokines, such as CXCL2/3/10/11 and CCL5, which were secreted in tumour tissues and recruited immunosuppressive cells, including Arg1^+^TANs and CD206^+^TAMs, that switched the microenvironment from immunoactive to immunosuppressive state. Evidence suggests that cancer cells actively modify the immune-microenvironment to promote tumour progression. For example, CRC cells acquire the immune regulatory molecules CD4 and CD45 to build a microenvironment suitable for tumour-infiltrating lymphocytes [33]. Hence, EGFR blockade induced stabilized GDH1 not only offered metabolic advantage but also remodeled TIME for tumour evasion, resulting in tumour survival and metastasis.

KRAS mutations are present in approximately 40% of mCRC cases. Preclinical studies demonstrate current strategies combining cetuximab with KRAS G12C inhibitors (e.g., adagrasib, onvansertib) have improved disease control rates in mCRC and effectively overcome monotherapy resistance. However, the G12C mutation subtype accounts for only about 5% of all mCRC patients. Consequently, the majority of mCRC cases harboring non-G12C KRAS mutations or KRAS wild-type status still require effective KRAS inhibitors to achieve synergistic effects with EGFR-targeted agents [34–36]. In accordance with clinical guidelines, the patient-derived samples analyzed in this study were restricted to KRAS wild-type cases. However, to dissect the potential universal role of GDH1 in adaptive resistance, we employed cellular models encompassing key genetic backgrounds for functional validation. Notably, the cetuximab tolerance conferred by GDH1 stabilization was consistently observed in KRAS wild-type, KRAS-mutant (e.g., G13D), and BRAF V600E-mutant cells (Figs. 2J and S2H), suggesting that this adaptive reinforcement mechanism may operate broadly across both KRAS wild-type and mutant contexts. Our study revealed that following EGFR restriction, the cytosolic accumulation of GDH1 epigenetically drives the stabilization of *SOS1* mRNA. The resultant abundance of SOS1, acting as the guanine nucleotide exchange factor (GEF) substrate for KRAS, enhances KRAS activity. Targeting the enzymatic activity of GDH1 disrupts this SOS1 upregulation, thereby limiting the elevation of KRAS activity under conditions of EGFR restriction. R162 is a tool compound with known off-target mitochondrial effects. While its use here supports the concept of targeting the GDH1/αKG axis, the observed phenotypes may not be solely attributable to GDH1 inhibition. Therefore, the translational potential of R162 itself is limited. Our genetic experiments (e.g., GDH1 depletion, Y451F mutation) provide more specific evidence for the on-target role of GDH1 in this adaptive pathway. However, R162 exhibits significant toxicity. Although this study utilized a low dose of R162 to induce GDH1 inhibition, we still cannot rule out the potential for metabolic toxicity in humans. Concurrently, αKG, a pivotal metabolite linking the TCA cycle and glutamate metabolism, is indispensable in normal tissues. This is particularly relevant given recent reports identifying αKG as a metabolite required for intestinal tissue regeneration [37]. Therefore, a critical future research direction is determining how to achieve moderate and appropriate GDH1 inhibition—abolishing its enhanced activity in tumor tissues while preserving its capacity to generate αKG in normal tissues.

In summary, cetuximab induced EGFR blockade, which stabilized GDH1, activating the downstream αKG/ALKBH5 axis. This leads to increased metabolic advantage and fosters an immunosuppressive tumor microenvironment through parallel, coordinated programs of OXPHOS and TIME remodeling. More importantly, αKG changes in blood may guide further immunotherapy.

## Materials and methods

### Clinical specimens

These clinical specimens were collected from the cohort of 49 patients with colorectal cancer who underwent diagnosis and treatment at Shanghai Tenth People’s Hospital of Tongji University in Shanghai, China. In accordance with contemporary clinical guidelines, tumor tissues from all patients were confirmed to be wild-type for both KRAS and BRAF genes prior to treatment initiation. Following surgical intervention and cetuximab therapy, these patients received systemic chemo- or immunotherapy. For those patients who experienced disease recurrence, pathological tissues were obtained through surgical resection or biopsy, and related follow-up information was gathered. All these samples were collected between Jan 2016 and January 2021. The clinic-pathological information of these patients is presented in Supplemental Table 1. The use of clinical samples was approved by the institutional review board of the hospital. All clinical samples were collected with informed consent under Health Insurance Portability and Accountability Act (HIPAA)-approved protocols.

### Experimental mice and in vivo treatment

All animal studies were conducted using male mice aged two or six weeks. The mice were group-housed (4–5 mice per cage), except for less than 5% of the mice, which were single-housed later because of the death of their cage mates. All mice were maintained under a 12-h light/dark cycle with free access to water and standard mouse chow. All mice received human care, and all experimental procedures were approved by the Institutional Animal Care and Use Committee (IACUC).

The mouse model of colorectal cancer-liver metastasis was described in a previous study [38, 39] with some improvements. Briefly, we first tested the liver metastasis of MC38 cells by injecting 5 × 10^4^ MC38 cells with or without GDH1 into the spleen. After 1 week, cetuximab (50 μg/mice/day) was intraperitoneally administered for a continuous 5 days. To rationale the effect of GDH1 on CRC-liver metastasis, we injected MC38 cells into the spleen and then separated MC38 cells from livers for three times circulation to improve the adaptation of MC38 cells in liver. Then, 5 × 10^4^ liver adaptive MC38 cells were inoculated in the cecum wall of C57BL/6 mice. After about 2 weeks, liver metastasis occurred. Cetuximab (50 μg/mice/day) alone, or combined with R162 (5 mg/kg), was intraperitoneally administered twice a week for 2 weeks. The combination group with PD-1 antibody (100 μg/mice/day) was intraperitoneally injected twice a week for 2 weeks. After treatment for 1 month, the mice were euthanized, and the tumours were dissected. During the outcome assessment (e.g., tumor volume measurement and histological analysis), the investigators were not blinded to the group allocation.

For the xenograft study, 1 × 10^6^ MC38 cells were subcutaneously injected into the left dorsal area of randomized 6-week-old female C57BL/6J mice. A week after inoculation, cetuximab (50 μg/mice) alone, or with R162 (5 or 30 mg/kg), was administered twice for 1 week. 10 days after treatment, the mice were euthanized, and the tumours were dissected. The tumour size was measured on indicated days using calipers in two dimensions to generate a tumour volume using the following formula: 0.5 × (length × width^2^).

### Drug treatment in vitro

To test cell viability under the treatment with capecitabine (Cape, 1 μM in medium), 5-FU (2 μM in medium), oxaliplatin (Oxa, 1 μM in medium) or cetuximab (Cetu, 4 µg/1 ml medium for MC38 and 12 µg/1 ml medium for HCT116) in plates, and the treatment was executed for the indicated time points with or without R162 (1 or 10 μM in medium). All cell lines were regularly tested and confirmed to be free of mycoplasma contamination. Cell line authentication was performed via STR profiling.

### αKG determination

The adherent cells were digested by trypsin for 1–2 min, and the xenograft tumours were digested by collagenase Ⅳ for 30–60 min. Two hundred microliters of tumour cells were collected in an Eppendorf tube (500 µl) with a scale (the scale was also accomplished by pipettes in which 200 µl of water were drawn into an Eppendorf tube, and we marked the volume as 200 µl on the tube wall). For relatively measuring the level of intracellular αKG, we have no need to quantify the detailed volume of cells, but we collected the same number of cells. The fractions of mitochondria, nuclei and cytosol were determined, and the volumes of mitochondria, nuclei and cytosol were also measured as described above. Next, the concentration of αKG was measured using an αKG assay kit according to the manufacturer’s instructions.

### Tumour-infiltrating immune cell isolation and flow cytometry

Mice were euthanized and sterilized in 75% alcohol for 3 min. The tumours were dissected, perfused, dissociated and filtered through a 30 μm strainer. The suspensions were then centrifuged at 60 g for 1 min and cells in supernatant were collected. Then, the tumour tissue was minced and placed in a 15 ml centrifuge tube, and 8 ml of digestion solution was added and shaken at 37 °C for 1 hour. The samples were centrifuged at 600 × *g* for 8 min and the cell pellet was collected. Then the cell pellet was resuspended in 10 ml 37.5% percoll solution. The samples were centrifuged at 500 × *g* for 30 min at 22 °C, the supernatant was removed. Next, 1 ml of erythrocyte lysis solution was added to the cell pellet, and the sample pipetted evenly and placed on ice for 1–3 min to lyse the erythrocytes. The lysis was terminated by adding 9 ml of PBS and centrifuging at 600 × *g* for 8 min, and the cell pellet was collected. The cell pellet was resuspended in 250 µl PBS + 2% FBS solution. Single cell suspensions were blocked with anti-mouse CD16/CD32 antibodies (Thermo Fisher) for 15 min. Then the indicated antibodies and live/dead dye were added and cells were stained on ice for 30 min in the dark prior to flow cytometry analysis.

### Subcellular fractionation

To obtain subcellular fractions, MC38 or HCT116 cells were harvested and washed three times with cold PBS. The xenograft tumours were digested by collagenase Ⅳ for 30–60 min to separate the tumour cells. Cytosol-nuclei fractionation was performed first, depending on different osmotic pressure tolerances. Then, the mitochondrial fractions were prepared using a Mitochondria/Cytosol Fraction Kit (BioVision).

### In vitro kinase assay

His-GDH1 (including WT and Y451F mutant) was purified from BL21 (DE3) cells. GST-EGFR (672-1186 amino acids) was pulled down from HEK293T cells and washed twice with cold PBS with 2% NP40, removing the associated proteins of EGFR. 50 ng GST-EGFR (672-1186 amino acids) and 1 µg of His-GDH were incubated in 25 µl of kinase reaction buffer (CST, #9802) for 30 min. The reaction was terminated by adding sample buffer and boiling for 8 min at 95 °C with SDS-loading buffer. WB analysis was used to measure the level of GDH1 ubiquitination using the indicated antibodies.

### MeRIP-sequencing and MeRIP-qPCR

3 μg of rabbit anti-m6A antibody (ab208577, Abcam) were bound to 25 μl washed Protein G Dynabeads (10003D, Thermo Fisher) in IP buffer (150 mM NaCl, 0.1% NP-40, 10 mM Tris-Cl, pH 7.5) at 4 °C for 3 h and washed twice in 1x IP buffer. Total RNAs were extracted with Trizol reagent (Thermo Fisher Scientific) and DNA contamination was removed using the DNase and DNase Buffer in the PrimeScript RT Reagent Kit (RR037B, Takara). The RNAs were then fragmented with RNA Fragmentation Buffer (10 mM Tris-HCl, 10 mM ZnCl_2_) for 5 min at 70 °C, purified by ethanol precipitation and quality-controlled using the Bioanalyzer RNA 6000 pico assay (5067-1513, Agilent).

Equal amounts of RNA (5 μg for rRNA-depleted and polyA-selected RNA and 200 μg for total RNA) were added to the antibody-bound beads and incubated for 3 h at 4 °C in the presence of RNase inhibitor (R301, Vazyme). The bound RNA was washed twice with 1x IP buffer, twice with low salt IP buffer (50 mM NaCl, 0.1% NP40, 10 mM Tris-Cl pH 7.4), twice with high salt IP buffer (500 mM NaCl, 0.1% NP40, 10 mM Tris-Cl pH 7.4) and once more in 1x IP buffer. The RNAs were then eluted from the beads using the RLT buffer supplied in the RNeasy Mini Kit (74106, Qiagen) and purified with the DireCTzol RNA Miniprep Kit (R2050, Zymo Research) before proceeding to library preparation. If subsequent RNAs were then subjected to RT-qPCR analysis, the cDNA synthesis was performed using the QuantiTect Reverse Transcription Kit (205311, Qiagen). The list of qPCR primers used in MeRIP-qPCR was in the Resources Table. For library preparation and sequencing, 150 ng of input RNA and the immunoprecipitated RNAs were dephosphorylated using Antarctic phosphatase and subjected to T4 PNK treatment (M0201, New England Biolabs). Directional libraries were prepared using the VAHTS Small RNA Library Prep Kit for Illumina (NR801, Vazyme) according to the manufacturer’s instructions. Libraries were sequenced on an Illumina HiSeq 2500 platform, generating single-end reads. The analysis of sequencing data referred to the reported paper [40]. Briefly, adaptor sequences were trimmed off for all raw reads using the Cutadapt software (version 1.2.1). Reads less than 35 nt or containing an ambiguous nucleotide were discarded by Trimmomatic (version 0.30). The remaining reads were aligned to the genome using TopHat (version 2.0.9). Only uniquely mapped reads with mapping quality score ≥20 were kept for the subsequent analysis for each sample. Then, the m6A-enriched peaks in each m6A immunoprecipitation sample were identified by MACS2 peak-calling software (version 2.0.10) with the corresponding input sample serving as control. MACS2 was run with default options except for “-nomodel, -keepdup all” to turn off fragment size estimation and to keep all uniquely-mapping reads, respectively. A stringent cutoff threshold for *P* value of 1 × 10^−5^ was used to obtain high-confidence peaks. Each peak was annotated based on Ensembl gene annotation information by applying BEDTools’ intersectBed (version 2.16.2).

### Biochemistry assay of ALKBH5 activity in vitro

The reaction system contains the following components: GG(m6A) CU RNA, ALKBH5, KCl (100 mM), MgCl2 (2 mM), RNasin (0.2 U/μl, Promega), L-ascorbic acid (2 mM), αKG (150 μM), (NH_4_)_2_ Fe (SO_4_)_2_·6H2O (150 μM) and 50 mM of HEPES buffer (pH 7.0). The reaction was incubated at room temperature for 2.5 h, then stopped by adding 5 mM EDTA followed by heating at 95 °C for 7 min. RNA was digested by nuclease P1 and alkaline phosphatase, and then analyzed on a HPLC system equipped with an Acclaim 120, C18, 5 μm analytical column (4.6×150 mm) (059148, Dionex) eluted with buffer A (5 mM ammonia acetate) and buffer B (60% acetonitrile, 0.01% TFA, 5 mM ammonia acetate) with a flow rate of 1 ml/min at room temperature. The detection wavelength was set at 260 nm.

### PDX on humanized mice

For in vivo PDX tumor-growth experiments, frozen PDX tumours developed from metastatic tumors of colon cancer patients (no. CRC10079 and no. CRC103611, collected from Ruijin hospital according to blood αKG) were prepared in a volume of 10-20 mm^3^, and then subcutaneously implanted into the flanks of six-week-old sex matched huHSC-NCG humanized mice purchased from Gempharmatech Co., Ltd., following standard procedures. The mice were enrolled for treatment on Day 5 when average tumor sizes reached about 150 mm^3^. Mice bearing established tumors were first administered with R162 at 5 mg/kg daily by intraperitoneal injection for three days. Anti-PD-1 antibodies (BioXCell) were injected intraperitoneally at a dose of 100 ug/mice twice per week for one week. Control groups received 10 ml/kg of vehicle (0.5% HPMC 0.2% Tween 80 solution) and 100 ug isotype control antibody/mice (Bio X Cell). The tumor size was measured weekly for at least three weeks using calipers in two dimensions to generate a tumor volume using the following formula: 0.5 × (length × width^2^).

## Supplementary information

Supplementary Information

Supplementary Tables

Original Western Blots

## Data Availability

All data, including the WB, IF and statistical data, were released upon reasonable request for leader contact Xiongjun Wang. The scRNA-seq original data accession codes in NCBI: SRP372718. All the proteomic data are accessible in NODE (https://www.biosino.org/node) with the accession number OEP00006672.
