# Supplementary material for: α-ketoglutarate accumulation orchestrates immunosuppressive metabolic remodeling to drive cetuximab resistance in metastatic colorectal cancer
Source: Cell Death Dis. 2026 Jun 30;17(1):810. doi: 10.1038/s41419-026-09001-8 (PMC13586316; doi:10.1038/s41419-026-09001-8)
Supplement: Supplementary file 1 [file 41419_2026_9001_MOESM3_ESM.pdf]

α-Ketoglutarate Accumulation Orchestrates Immunosuppressive Metabolic Remodeling to Drive Cetuximab Resistance in Metastatic Colorectal Cancer

Supplemental Information

Original western blots

Figure S1

S1A

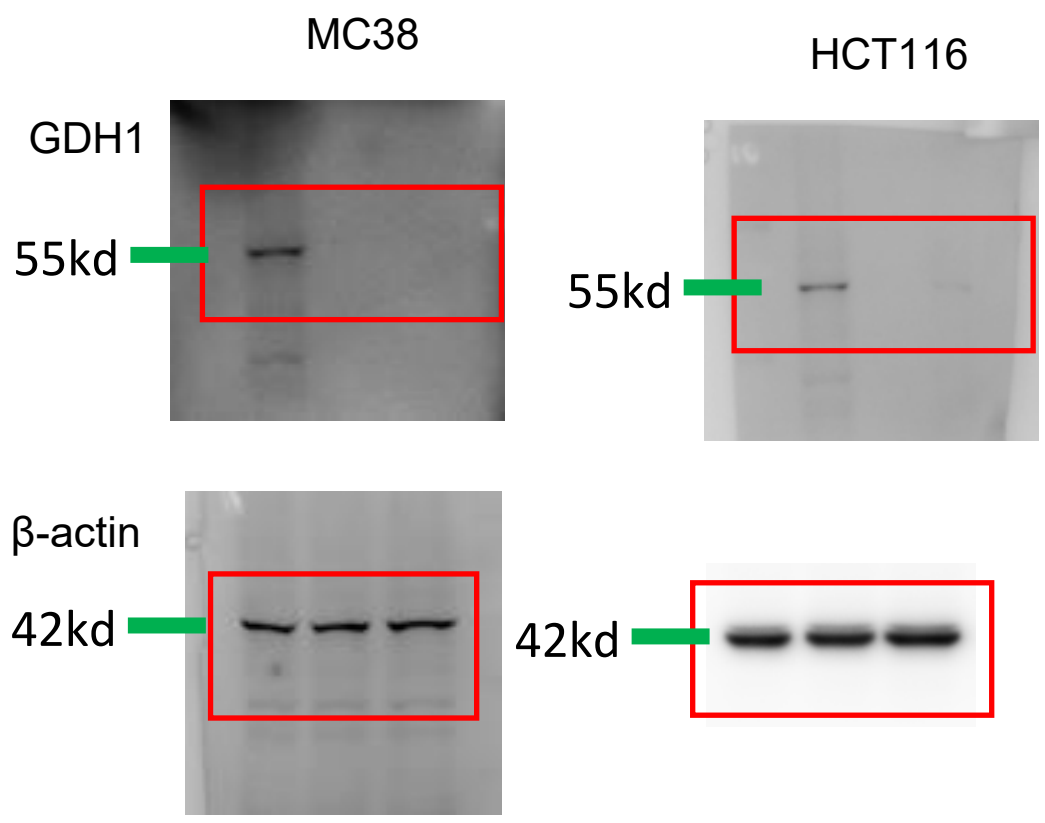

S1C

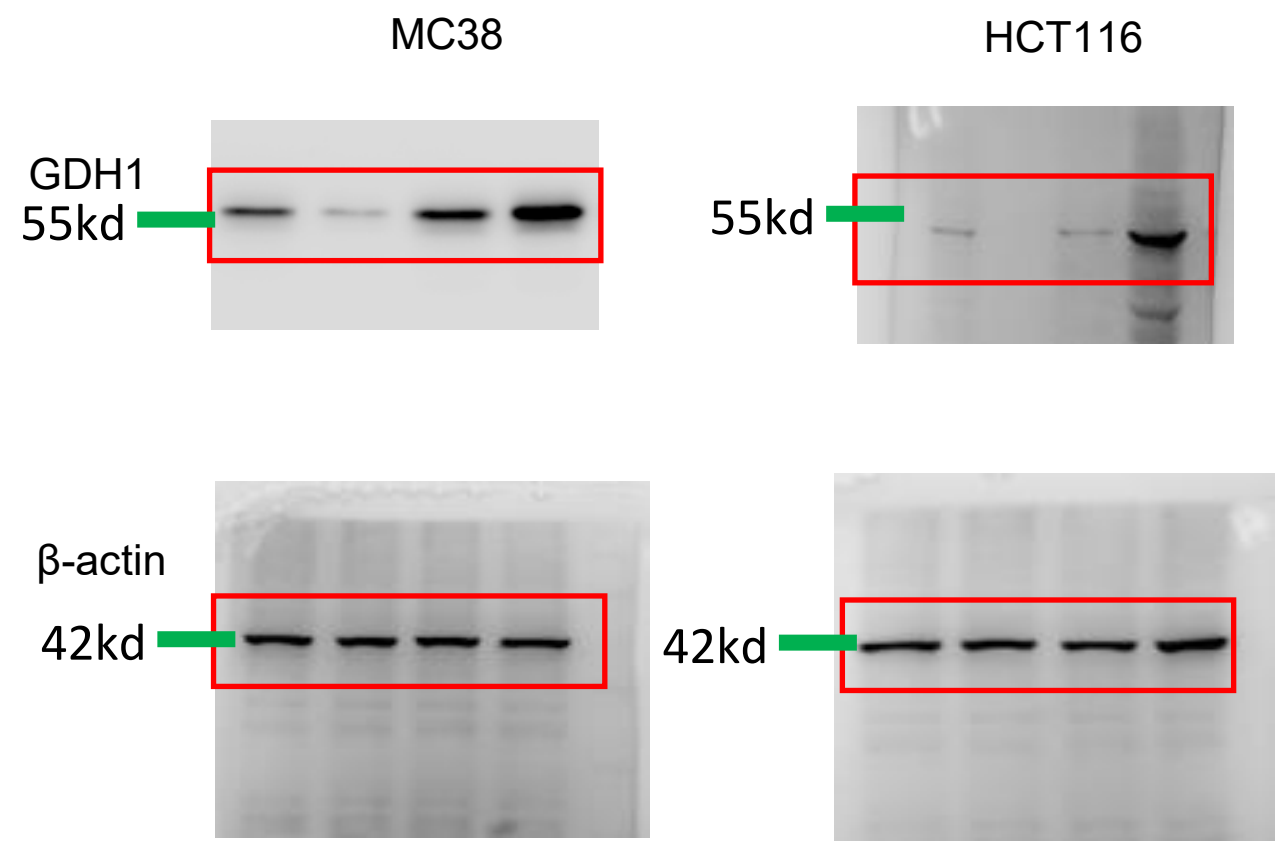

Figure 2

2A

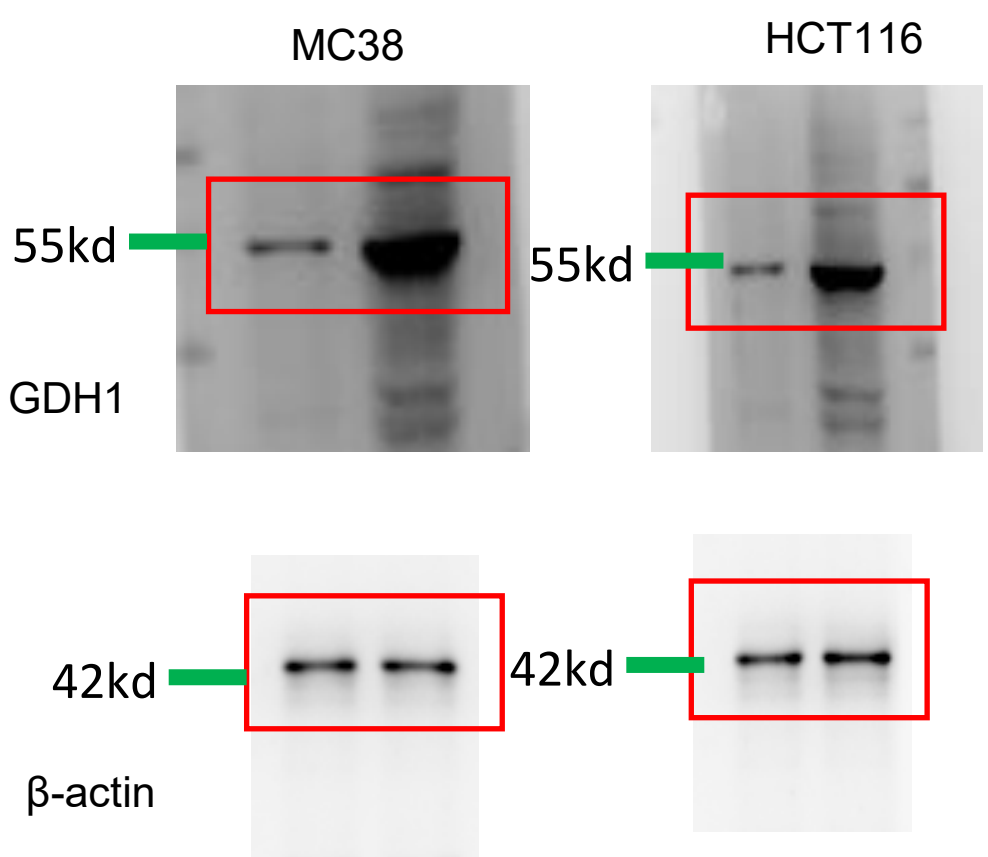

2C

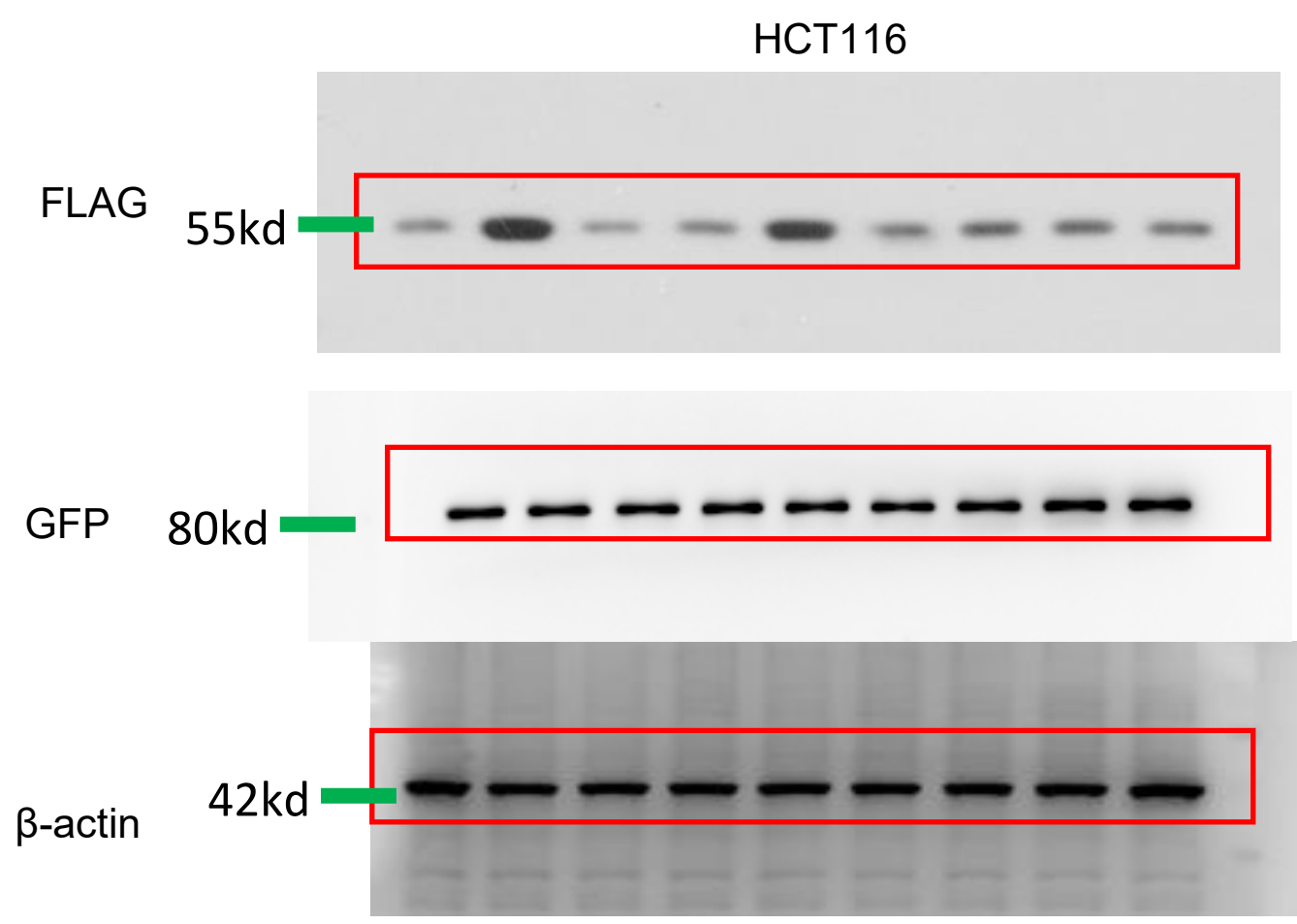

2D

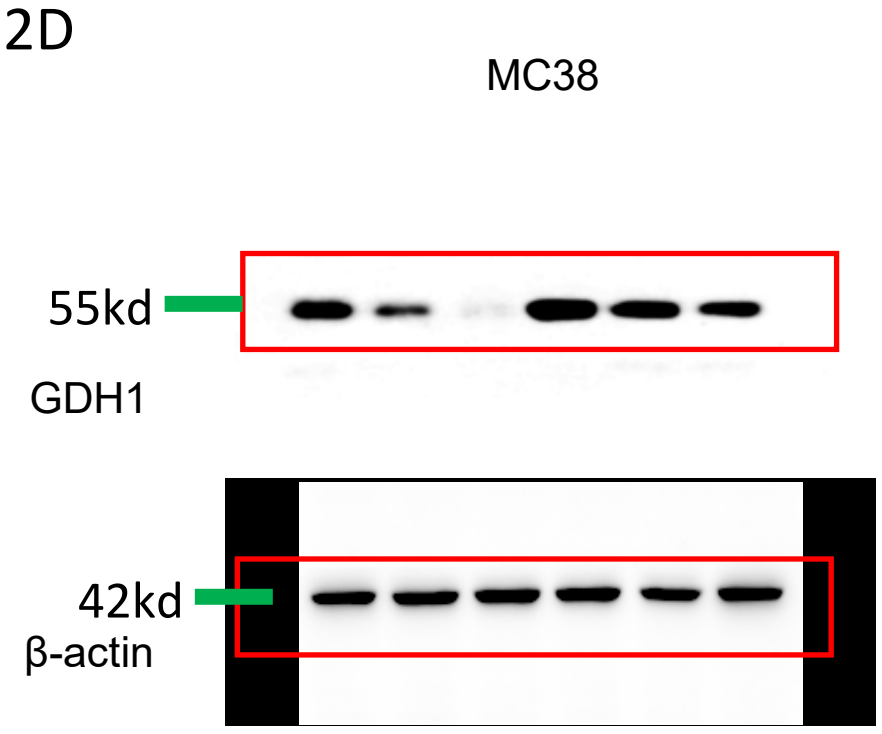

2E

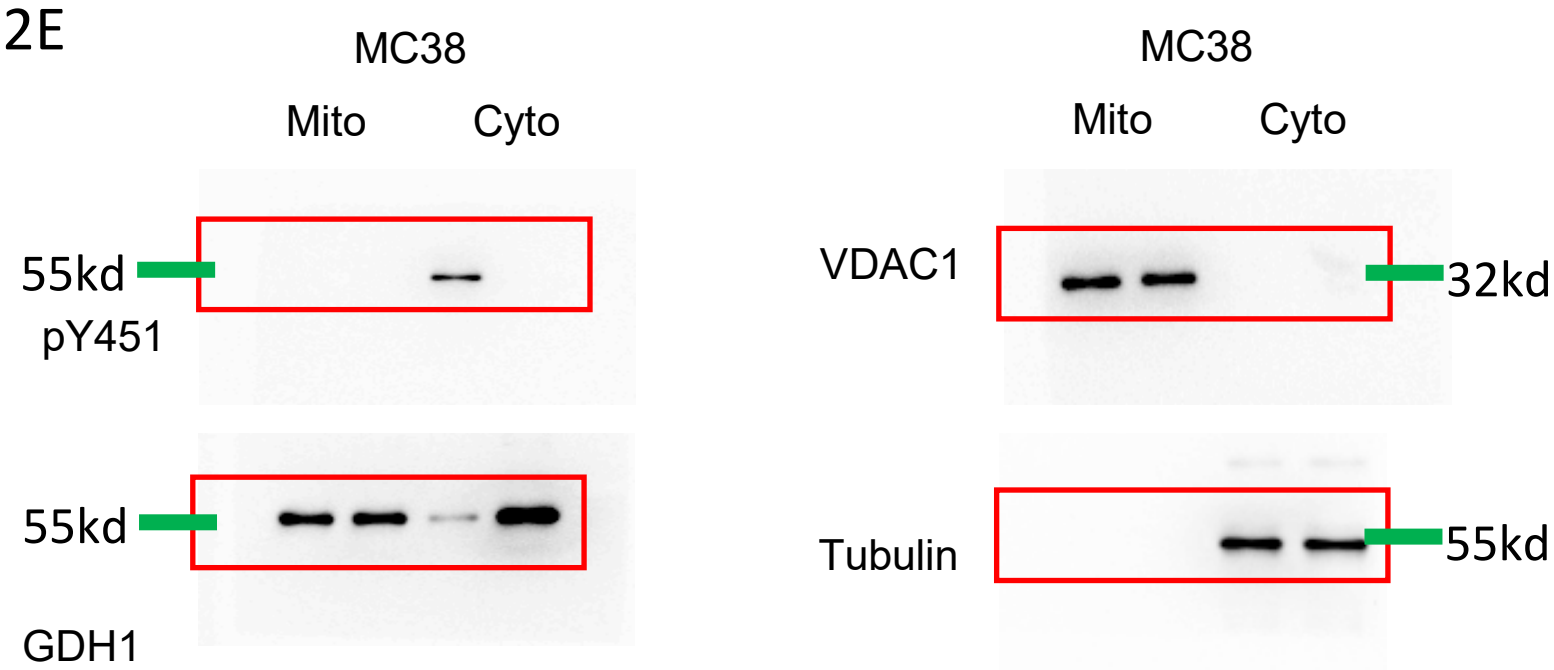

2G

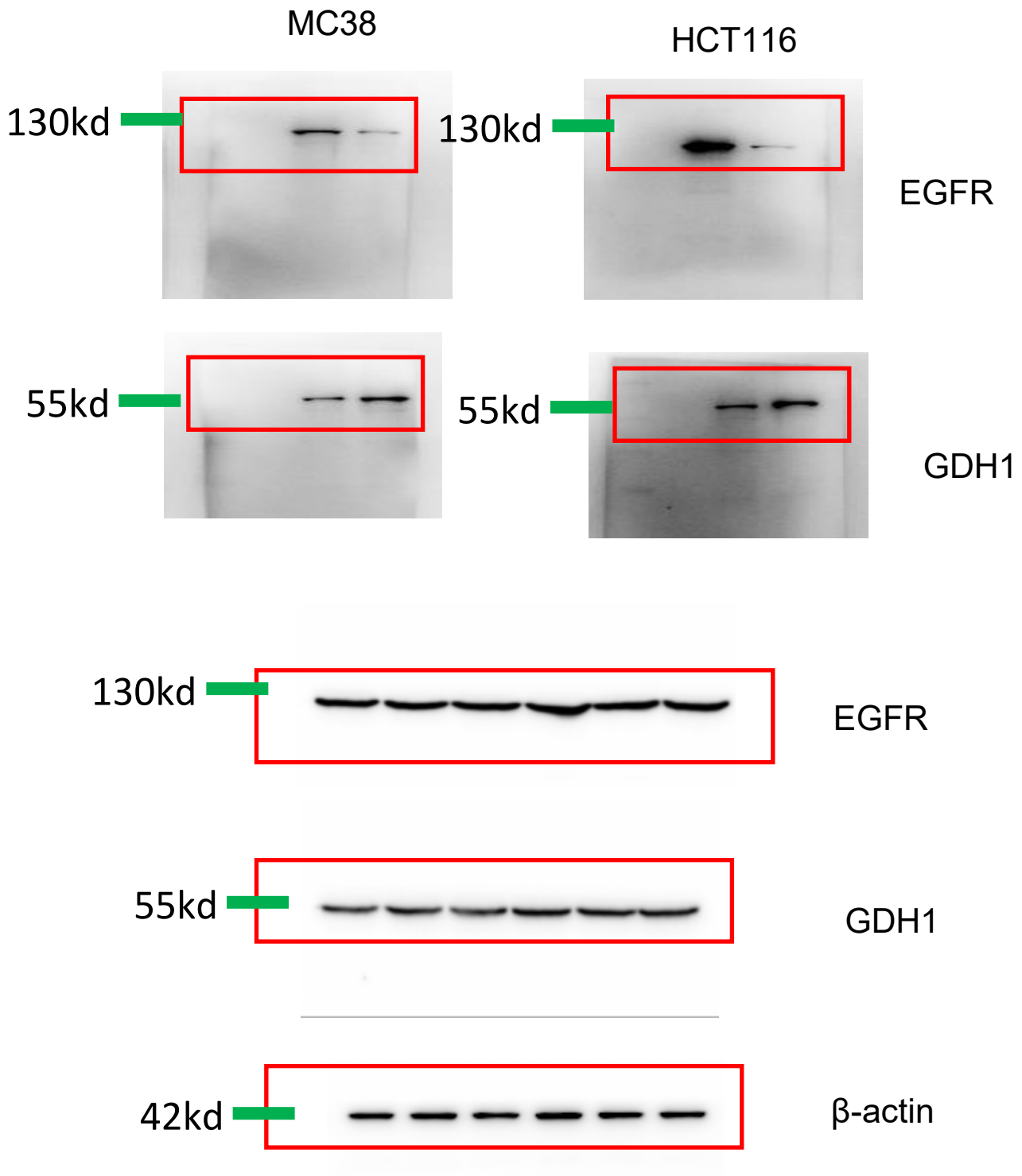

2H

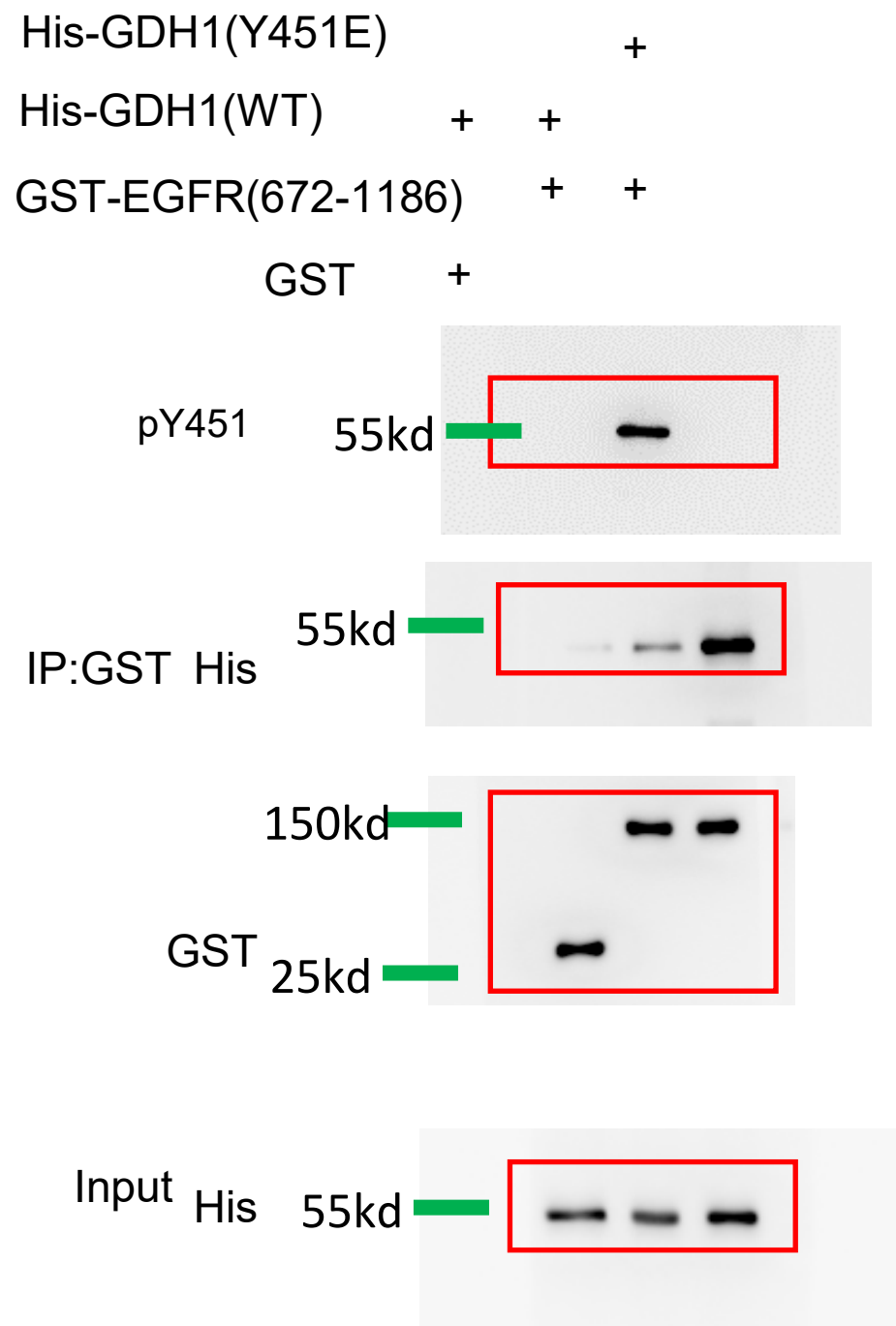

2I

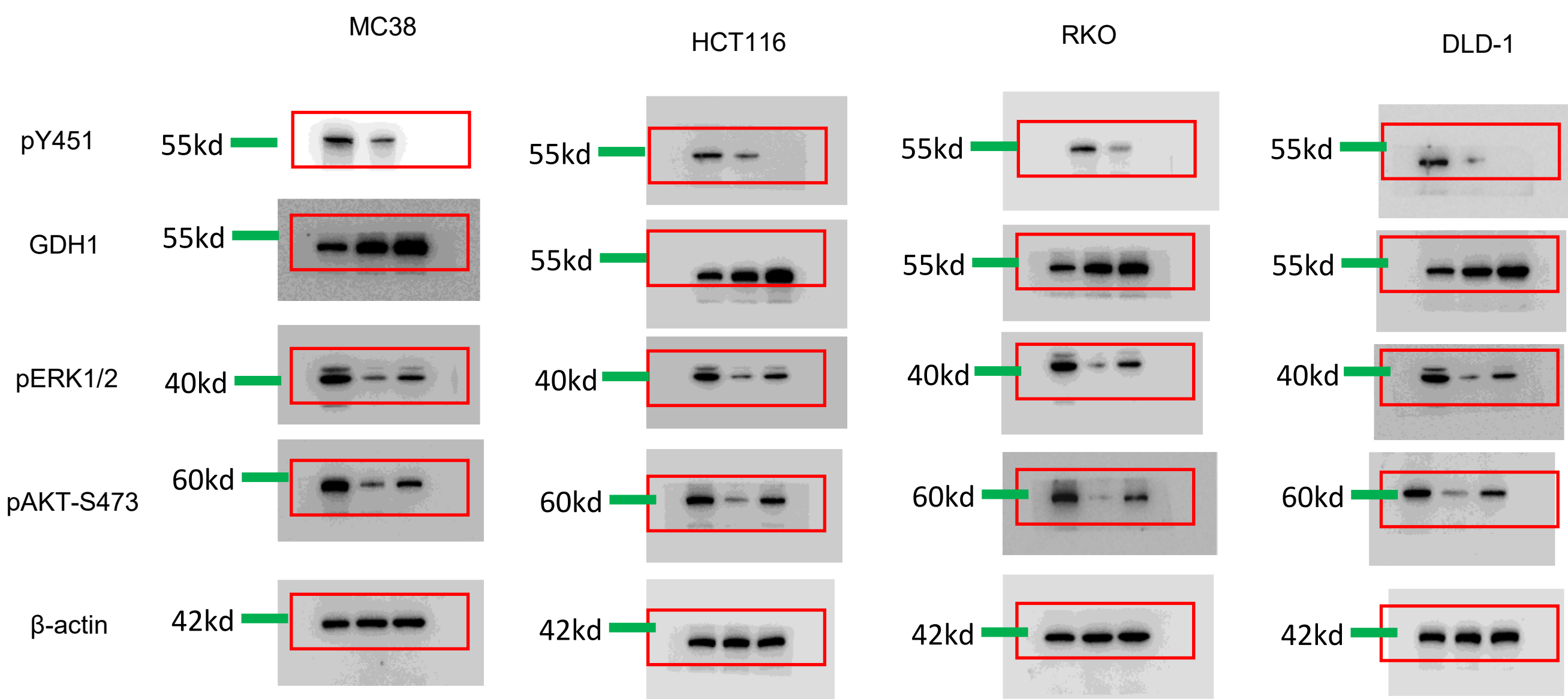

Figure 3

3A

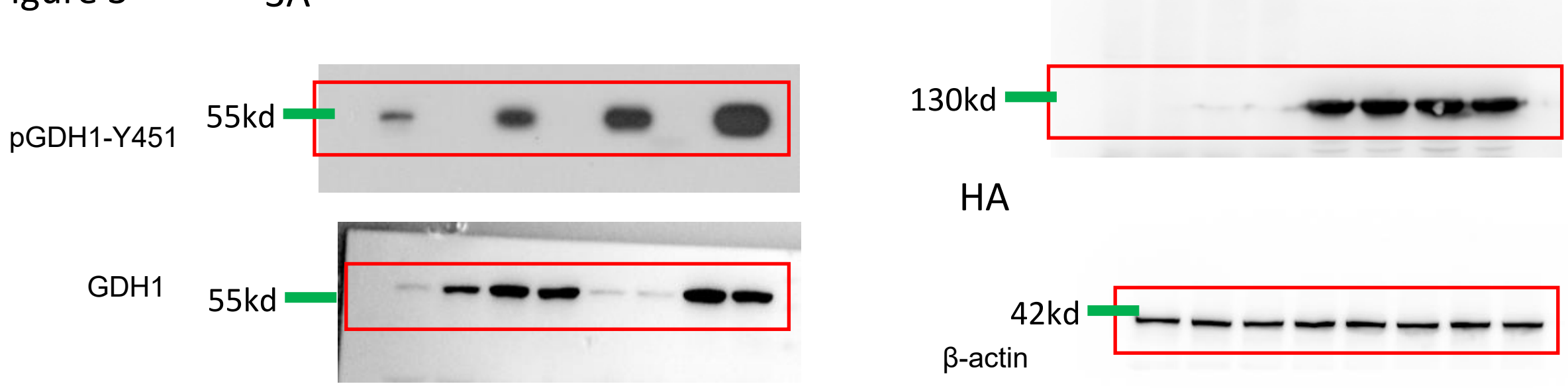

S2B

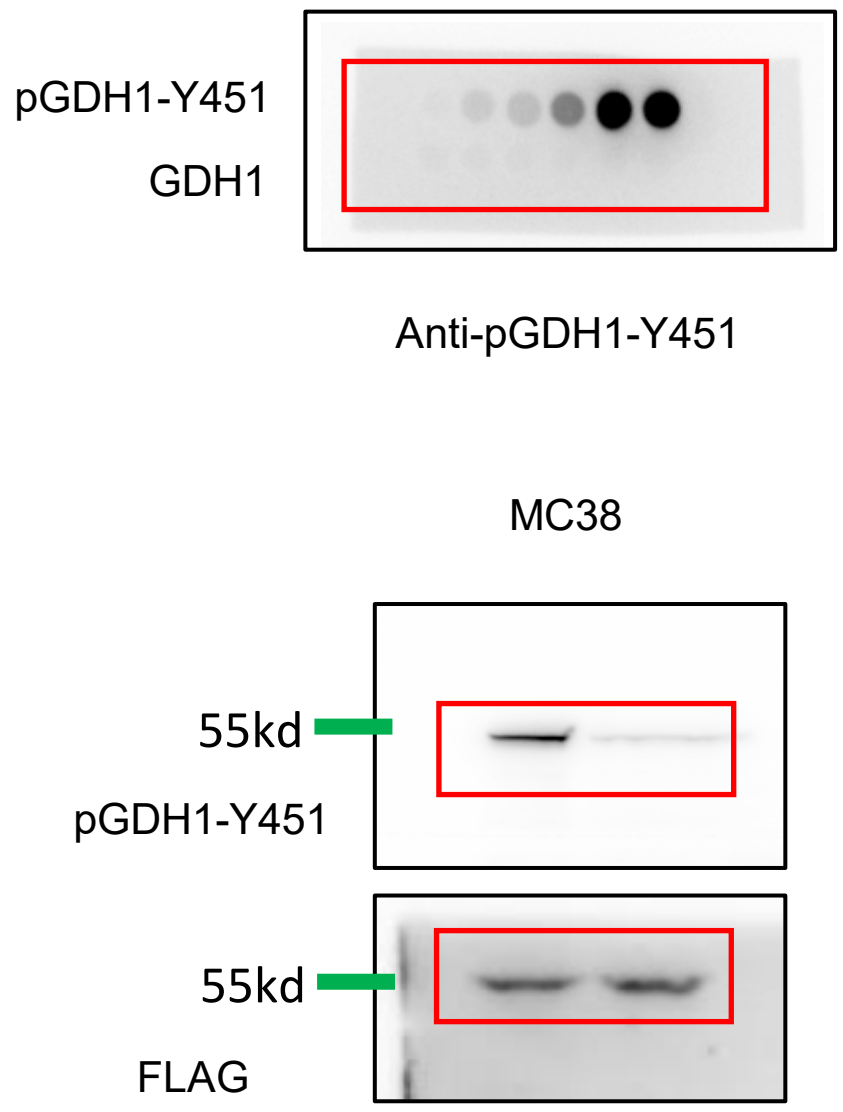

S2D

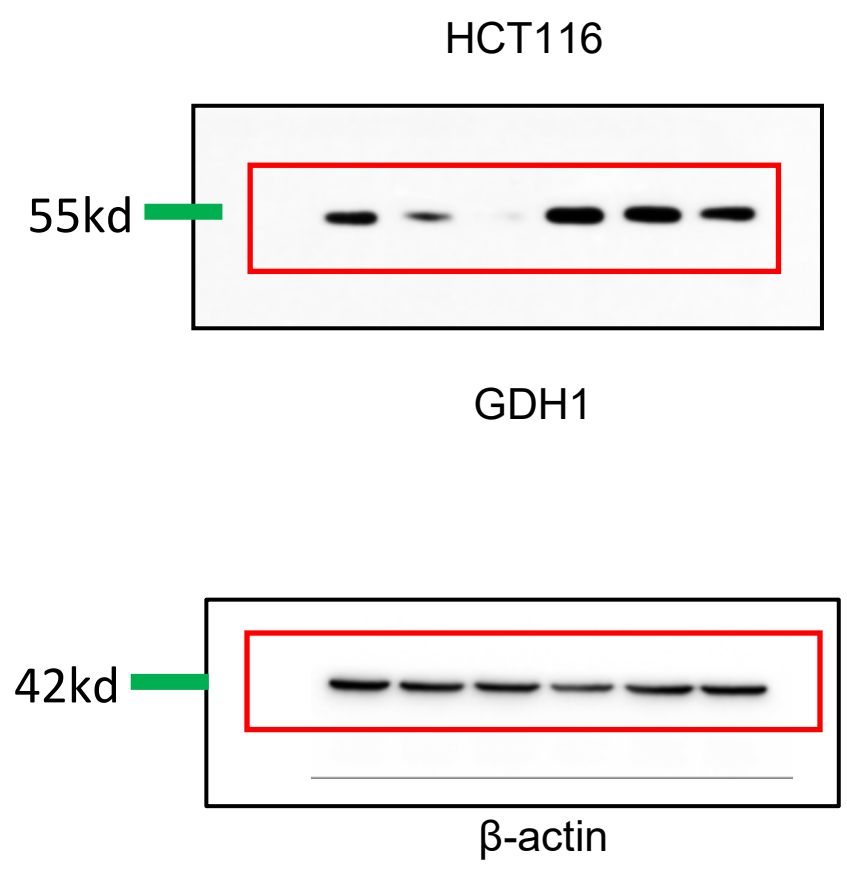

S2F

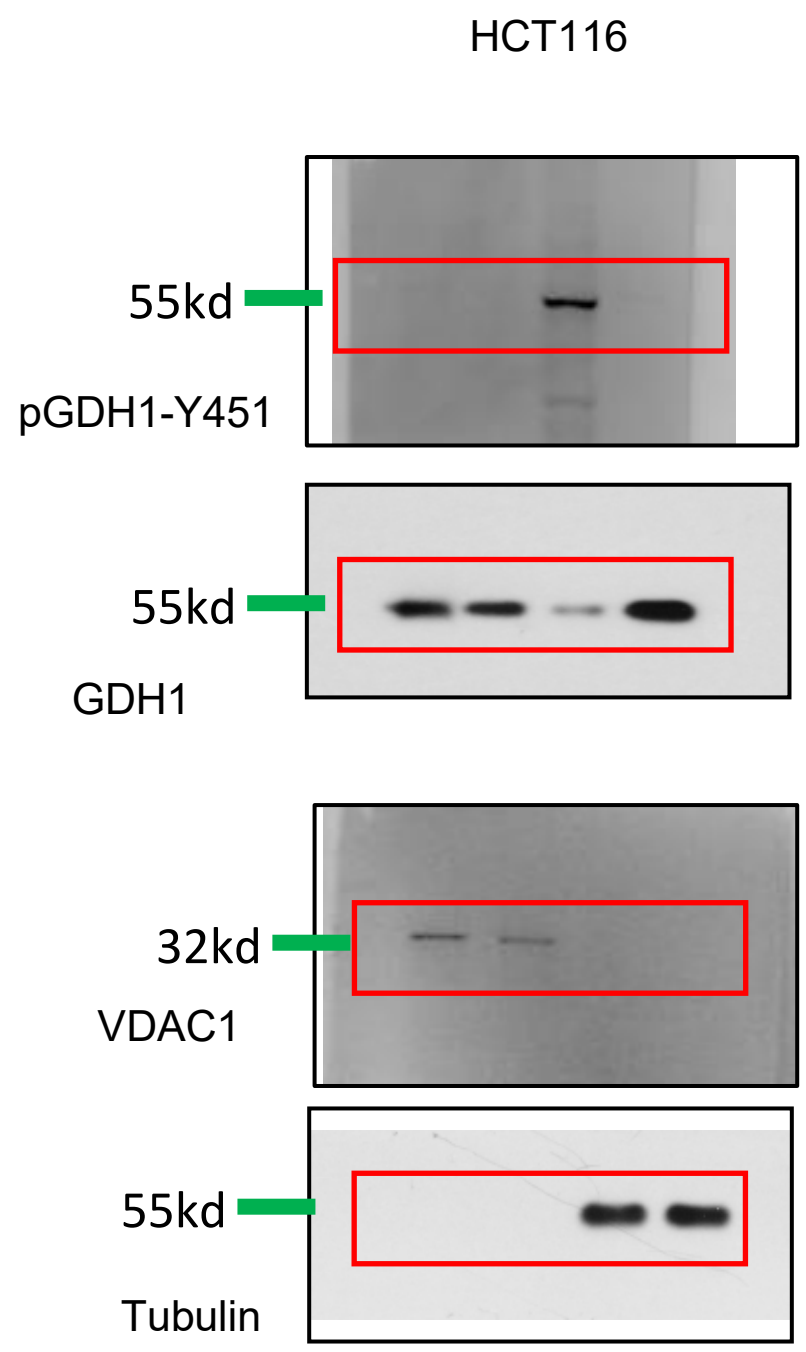

S2G

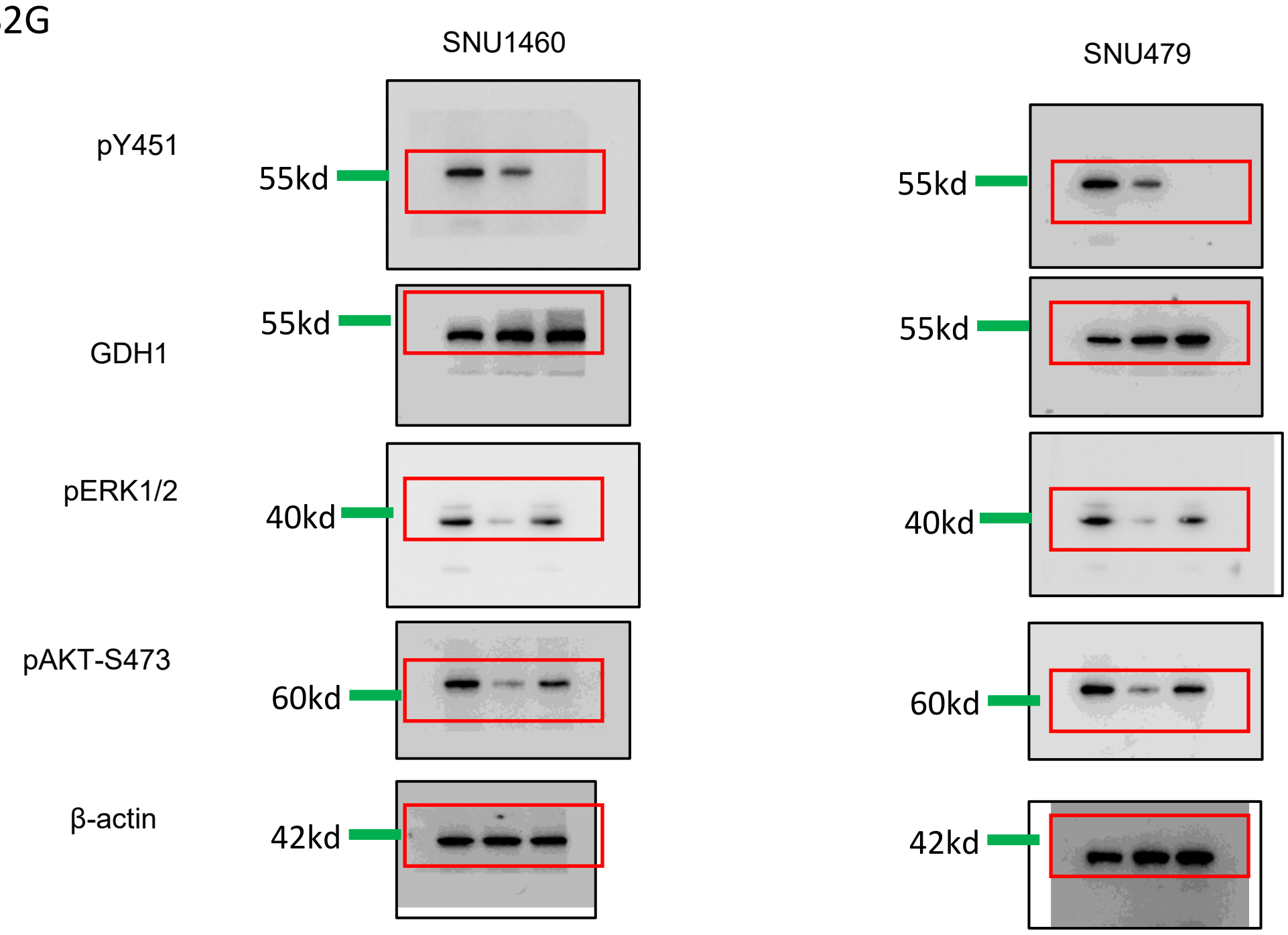

Figure 3

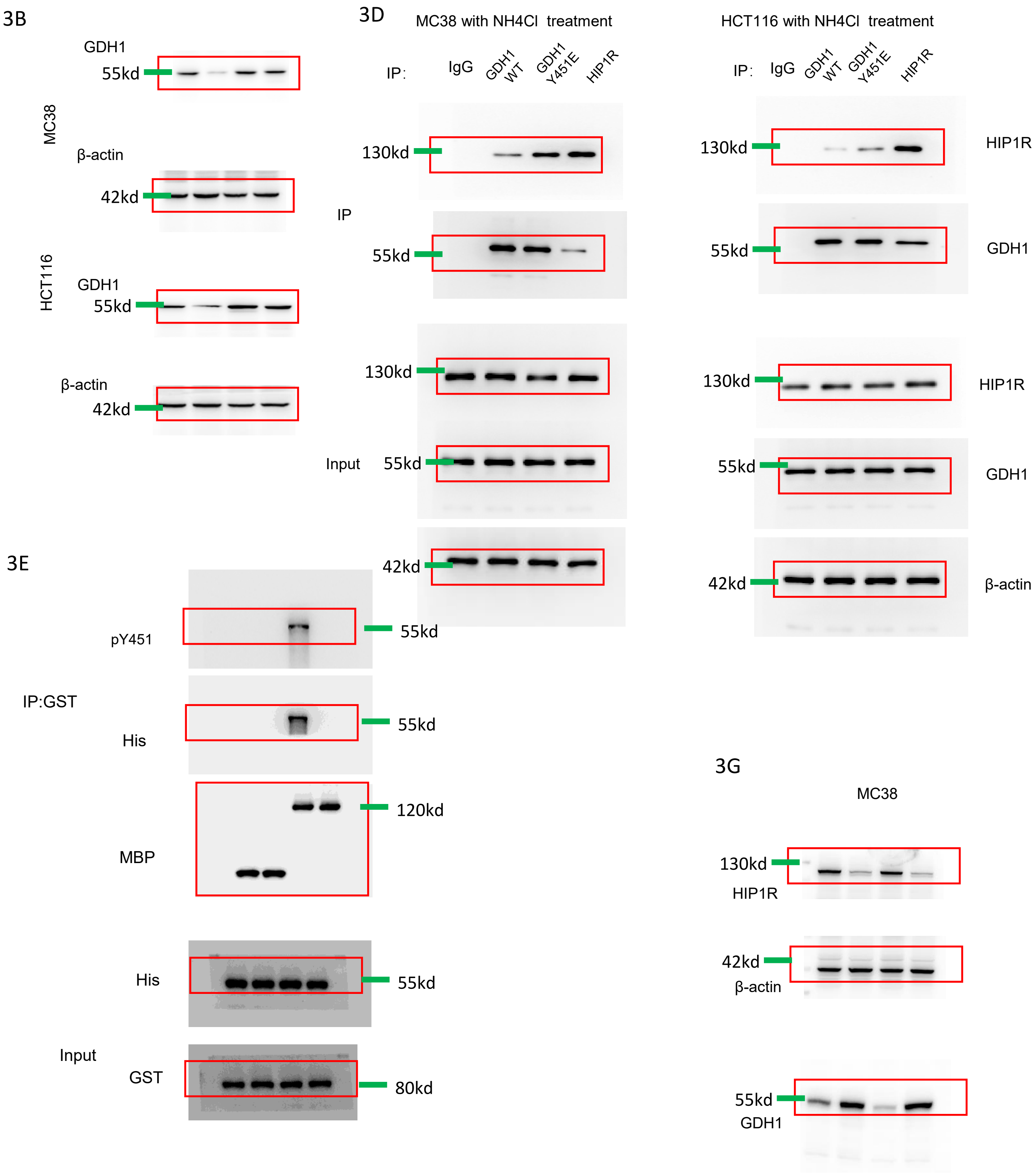
3B
GDH1
55kd
MC38
β-actin
42kd
HCT116
GDH1
55kd
β-actin
42kd
3D
MC38 with NH4Cl treatment
IP:
IgG
GDH1 WT
GDH1 Y451E
HIP1R
130kd
IP
55kd
130kd
Input
55kd
42kd
HCT116 with NH4Cl treatment
IP:
IgG
GDH1 WT
GDH1 Y451E
HIP1R
130kd
HIP1R
55kd
GDH1
130kd
HIP1R
55kd
GDH1
42kd
β-actin
3E
pY451
55kd
IP:GST
His
55kd
MBP
120kd
His
55kd
Input
GST
80kd
3G
MC38
130kd
HIP1R
42kd
β-actin
55kd
GDH1

S3A

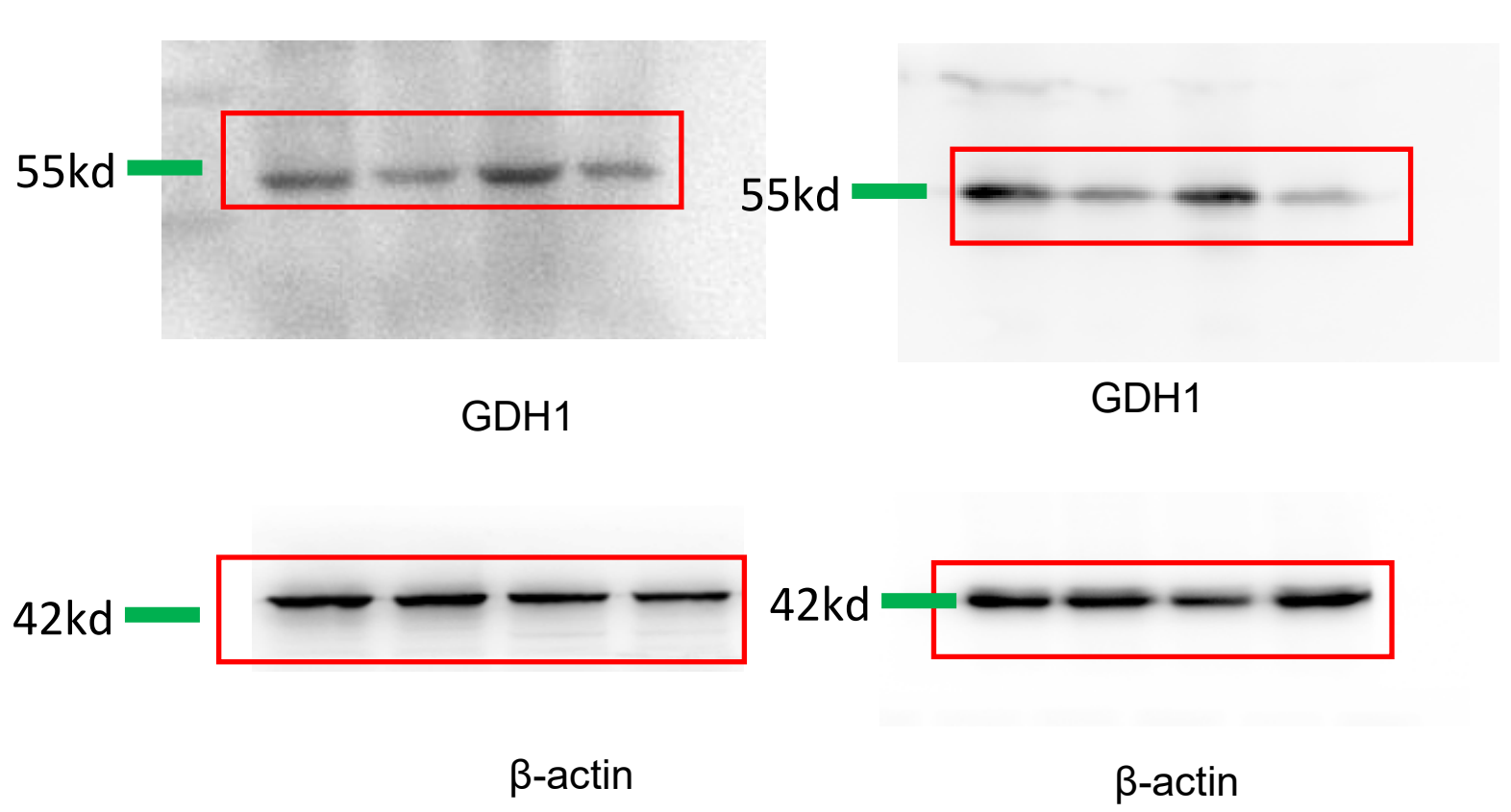

S3C

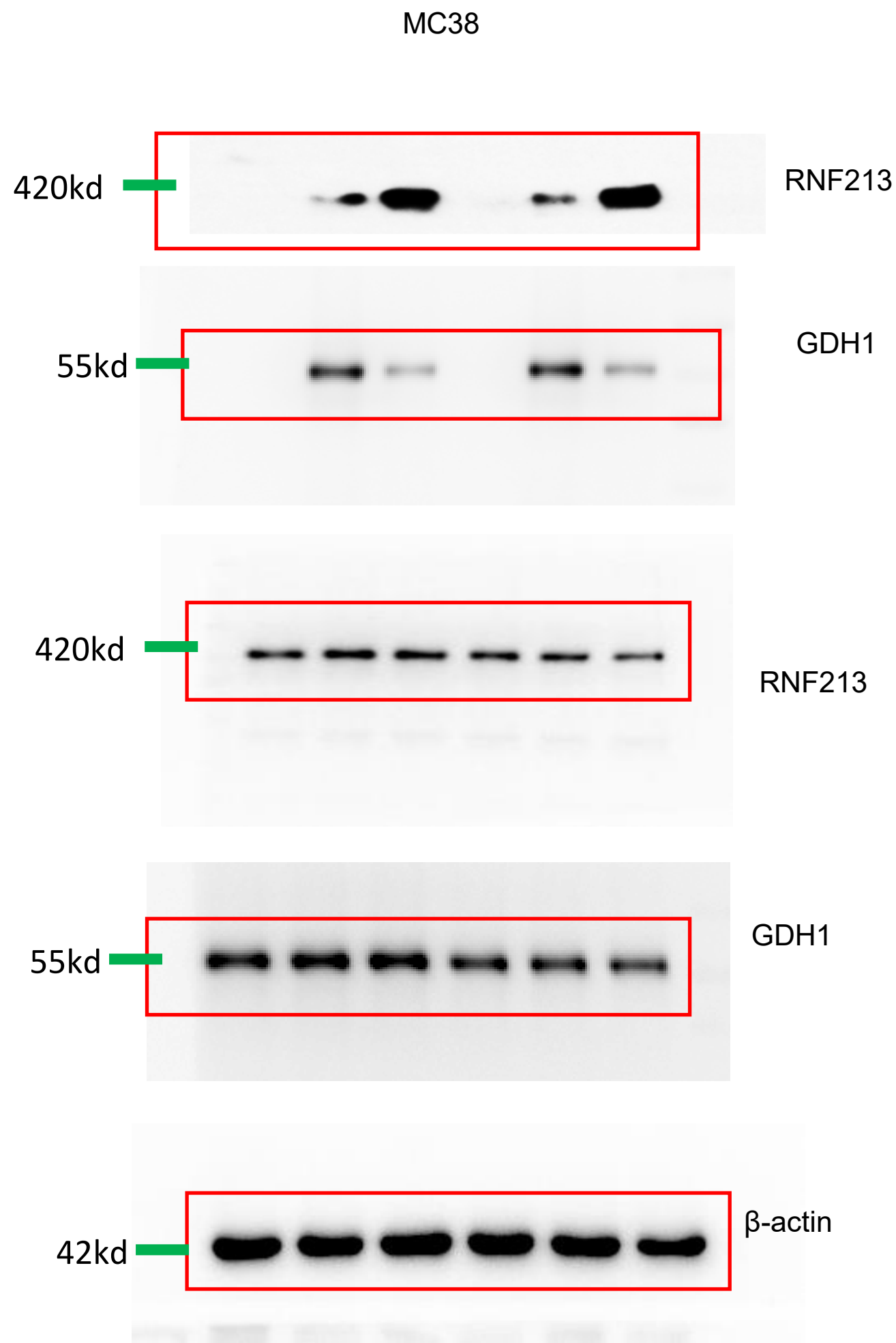

S3E

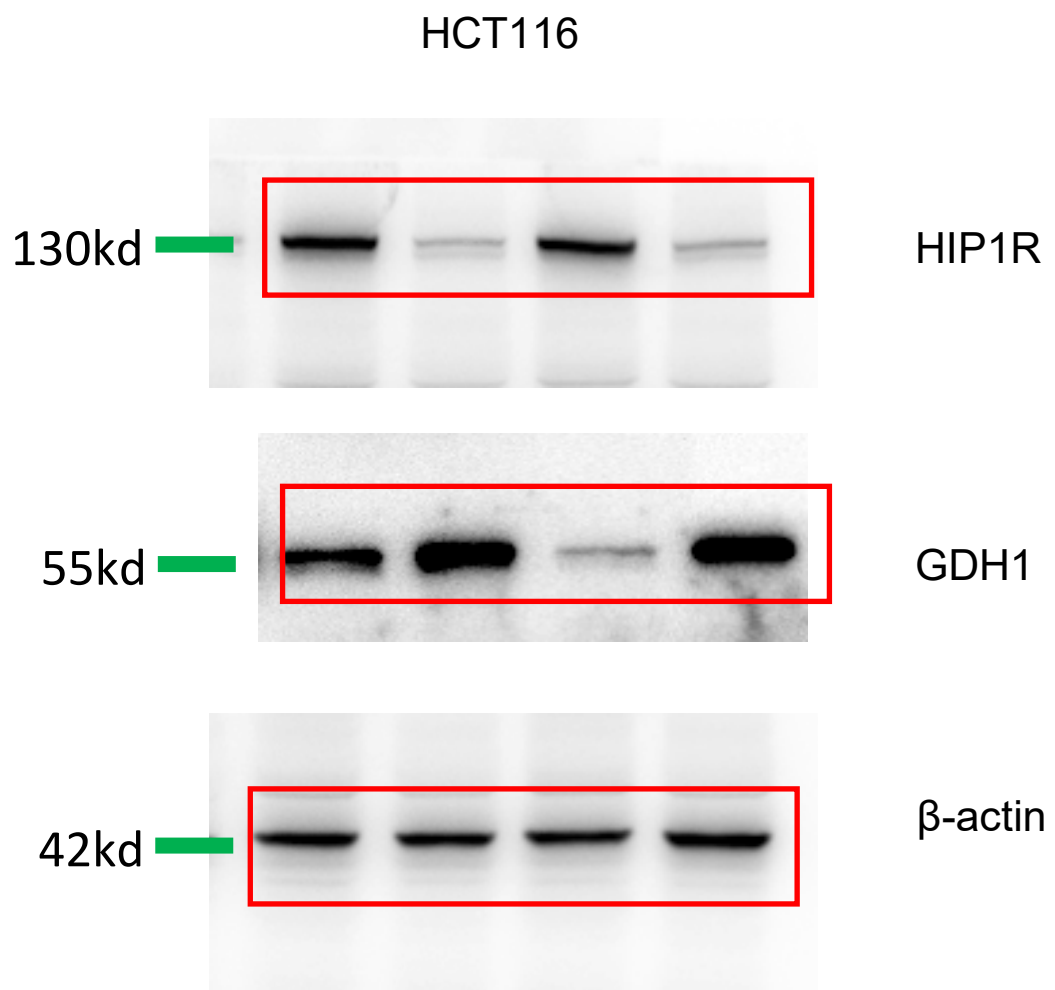

S3F

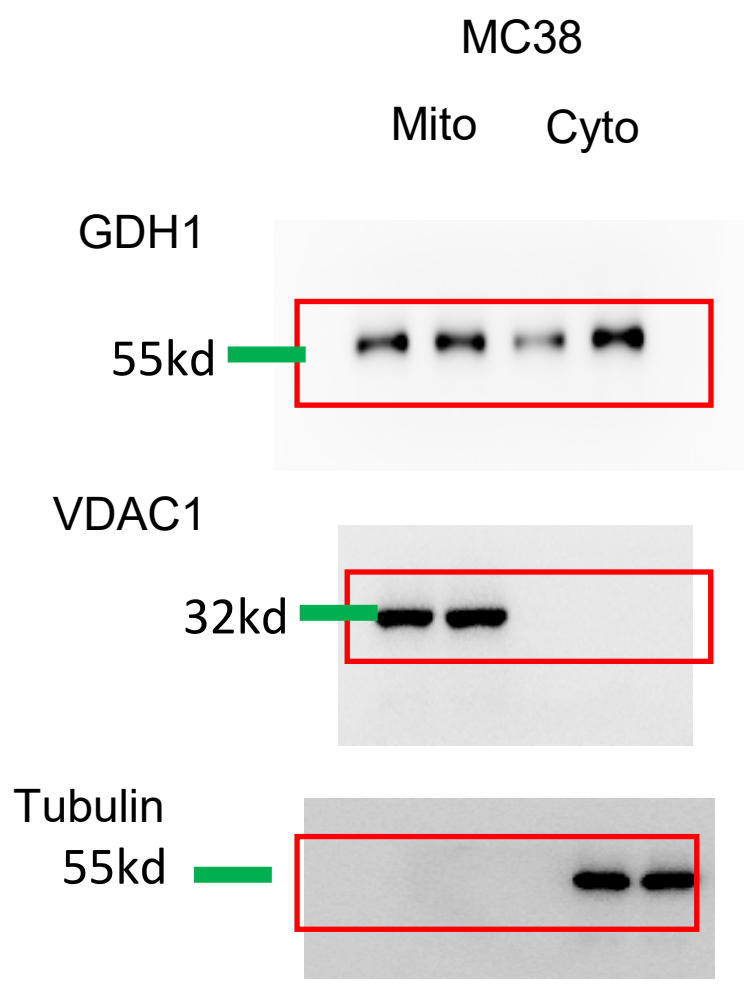

Figure 4

4D

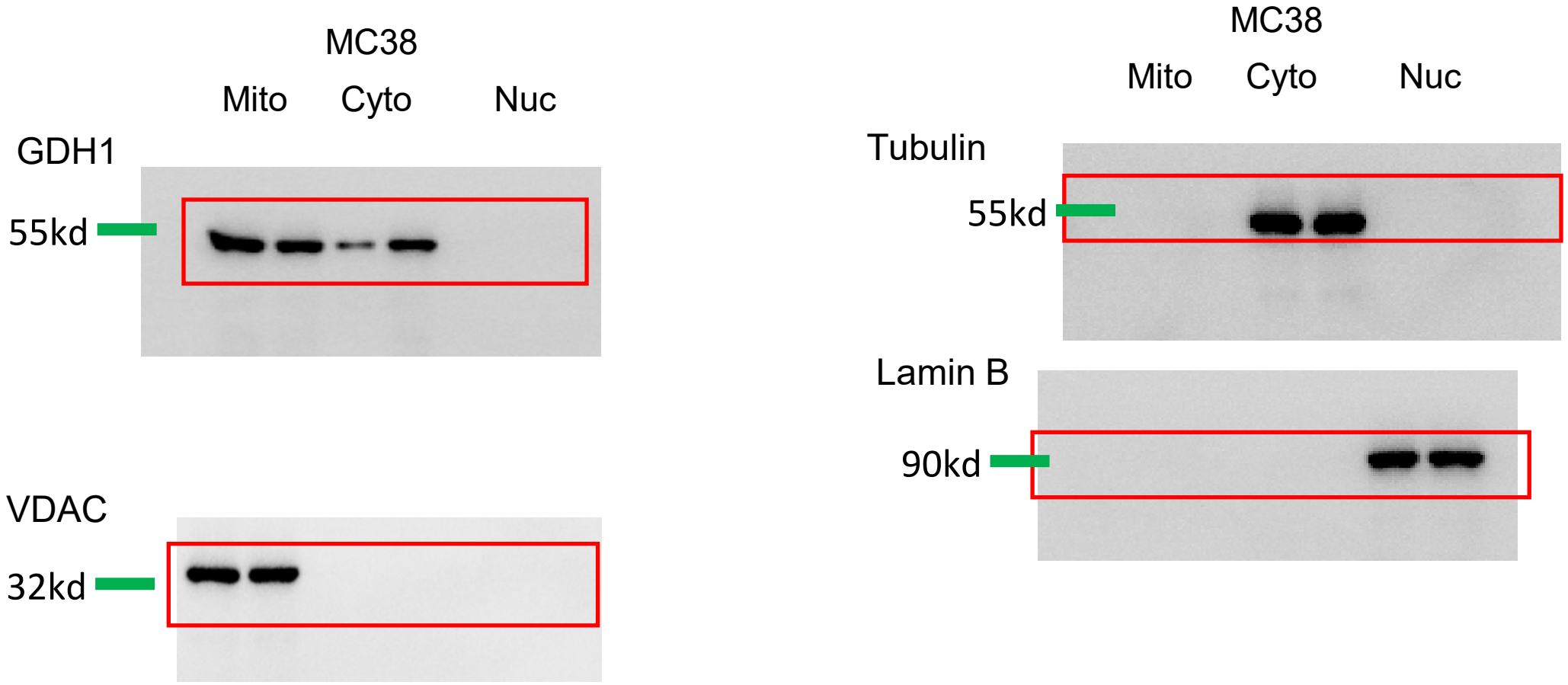

S4C

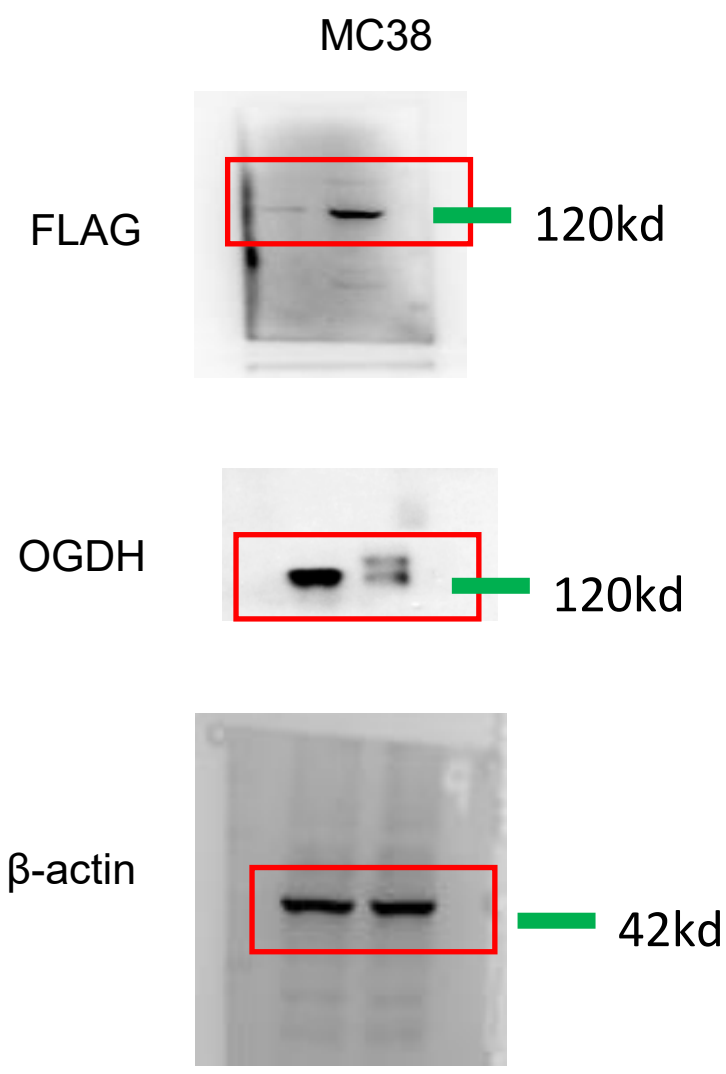

S4G

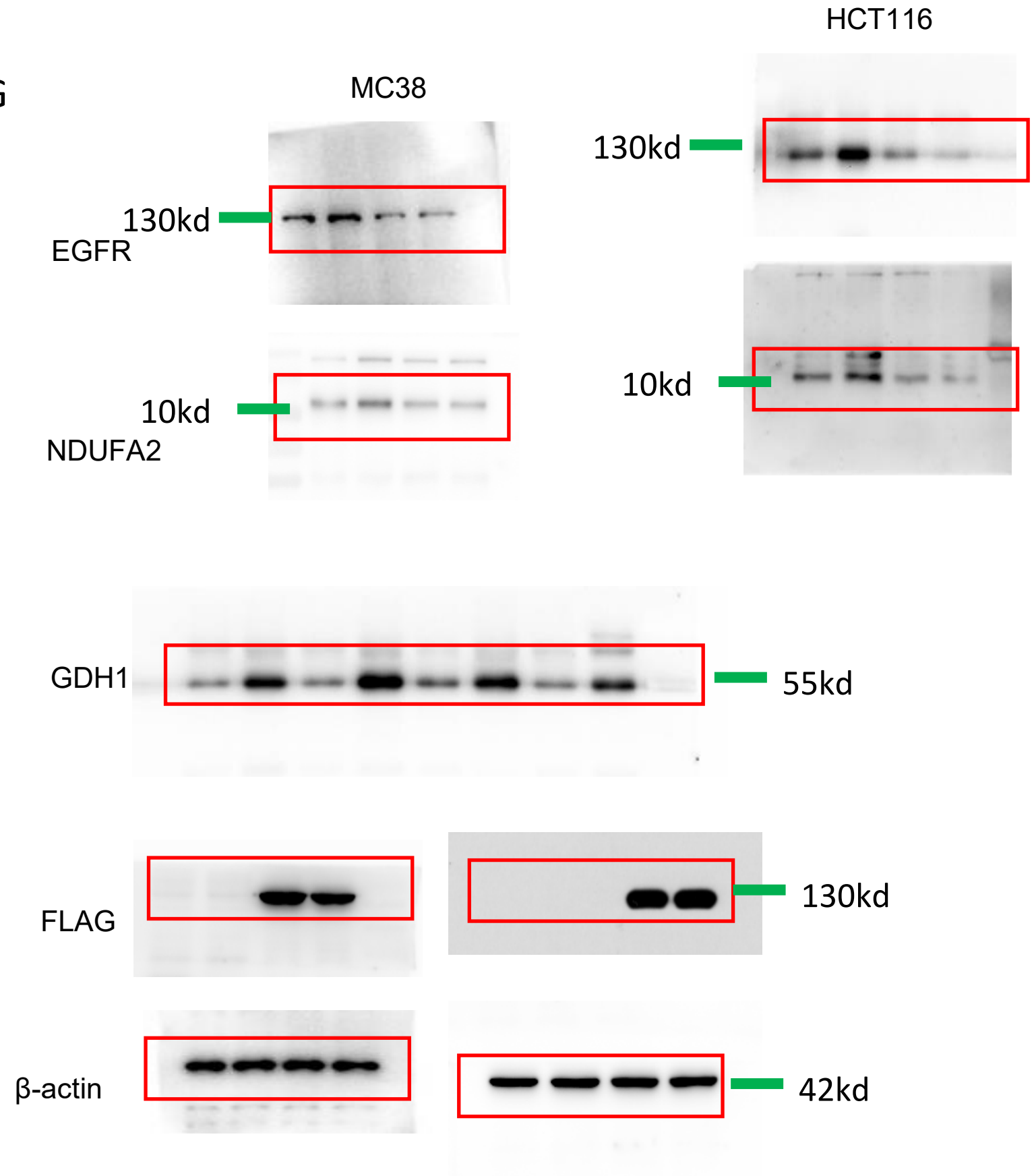

Figure 5

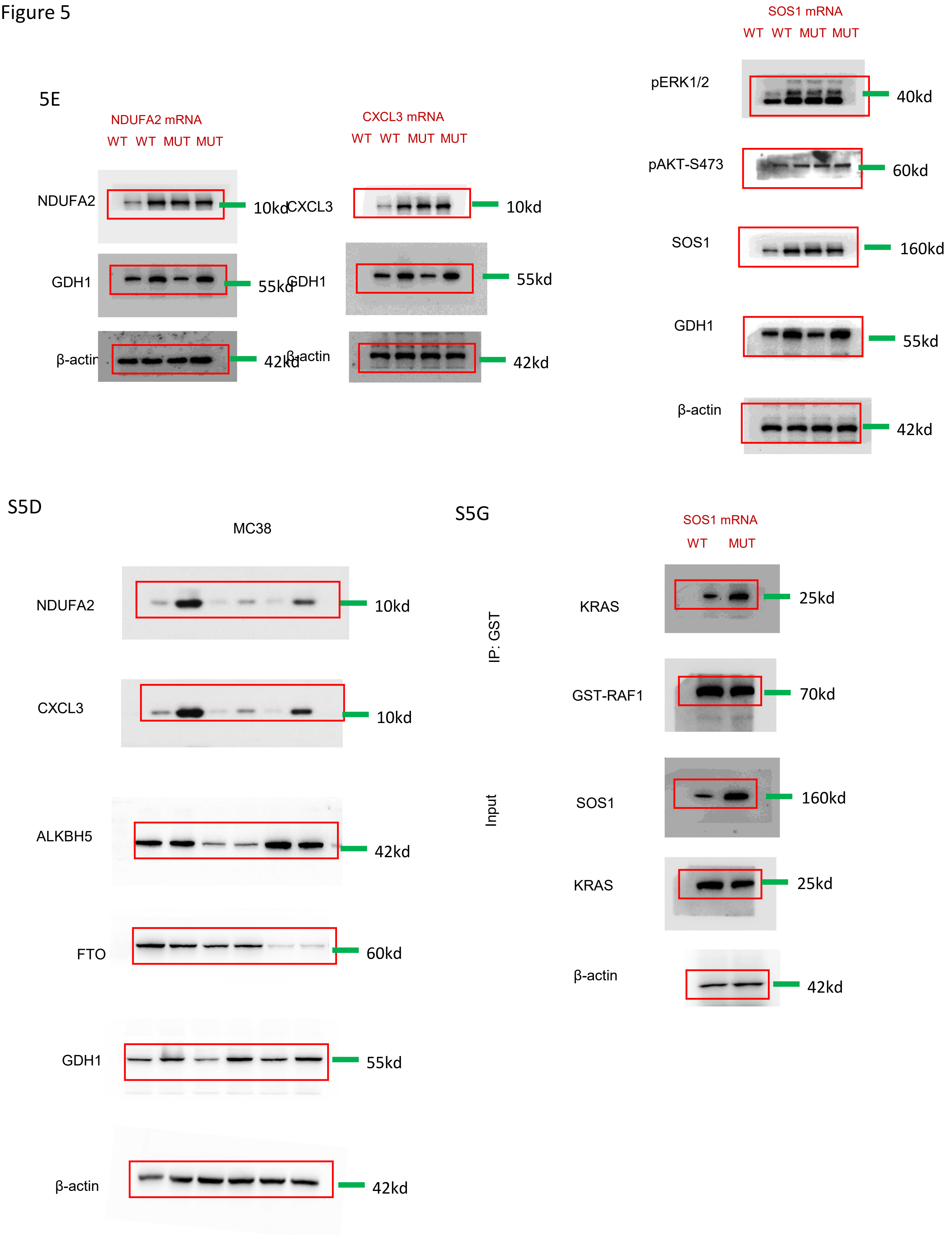
5E
NDUFA2 mRNA
WT WT MUT MUT
NDUFA2
10kd
GDH1
55kd
β-actin
42kd
CXCL3 mRNA
WT WT MUT MUT
CXCL3
10kd
GDH1
55kd
β-actin
42kd
SOS1 mRNA
WT WT MUT MUT
pERK1/2
40kd
pAKT-S473
60kd
SOS1
160kd
GDH1
55kd
β-actin
42kd
S5D
MC38
NDUFA2
10kd
CXCL3
10kd
ALKBH5
42kd
FTO
60kd
GDH1
55kd
β-actin
42kd
S5G
SOS1 mRNA
WT MUT
IP: GST
KRAS
25kd
GST-RAF1
70kd
Input
SOS1
160kd
KRAS
25kd
β-actin
42kd

Figure 6

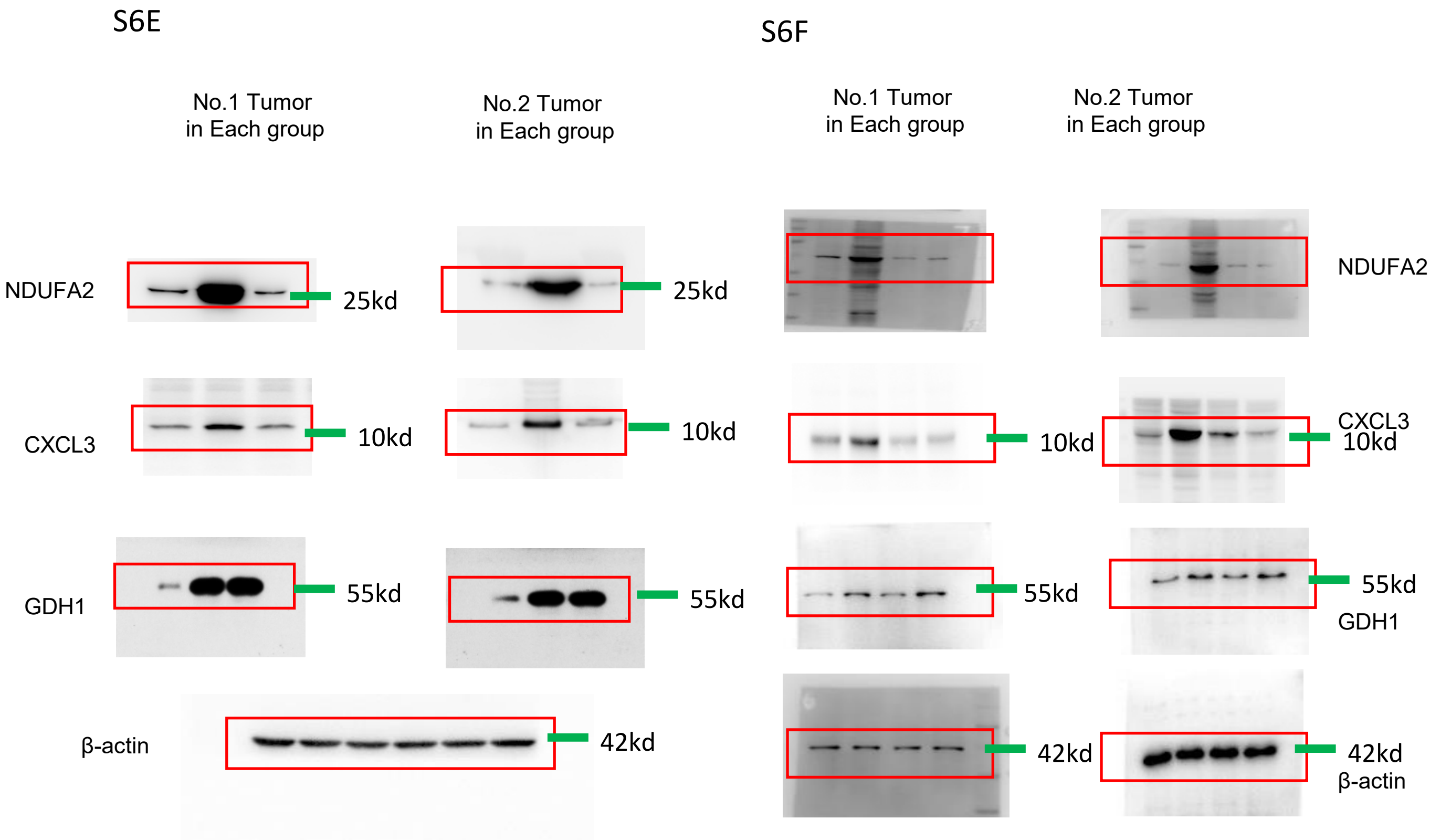
S6E
S6F
No.1 Tumor in Each group
No.2 Tumor in Each group
No.1 Tumor in Each group
No.2 Tumor in Each group
NDUFA2
25kd
25kd
NDUFA2
CXCL3
10kd
10kd
10kd
CXCL3
10kd
GDH1
55kd
55kd
55kd
55kd
GDH1
β-actin
42kd
42kd
42kd
β-actin
