# Supplementary material for: α-ketoglutarate accumulation orchestrates immunosuppressive metabolic remodeling to drive cetuximab resistance in metastatic colorectal cancer
Source: Cell Death Dis. 2026 Jun 30;17(1):810. doi: 10.1038/s41419-026-09001-8 (PMC13586316; doi:10.1038/s41419-026-09001-8)
Supplement: Supplementary file 2 [file 41419_2026_9001_MOESM1_ESM.docx]

**Supplemental Information**

**Supplemental Figures and Legends**

**Supplementary Figure 1.** (A-B) WB was performed to confirm the successful construction of GDH1 knockdown in MC38 or HCT116 cells (A); above cells were used to perform αKG determination (n=3, B). (C) MC38 or HCT116 cells stably expressing shNT or shGDH1 were treated with or without Cetu for the indicated times, and cell viability was assessed. n=3. (D) The schematic diagram of adapted MC38 cells for colon-liver metastasis. Briefly, we first tested the liver metastasis of MC38 cells by injecting 5 × 10^4^ MC38 cells into spleen, after 1 week, cetuximab (50 μg/mice/day) was intraperitoneally administered for continuous 5 days. Then we separated MC38 cells from successful liver metastasis. After three times circulation to improve the adaptation of MC38 cells in liver, 5 × 10^4^ liver adapted MC38 cells were inoculated in the cecum wall of C57BL/6 mice to obtain colon-liver metastatic CRC cells. (E) UMAP plots showing the expression of Ptprc in CD45^+/-^ immune cells. (F-G) Reclustering of the CD45^+/-^ immune cells in Mock or Cetu treatment group, represented as a UMAP plot. (H-I) The expression of characteristic genes in neutrophil and other immune cell clusters. (J) Flow cytometry (FCM) verified the percentage change of TAMs (total and M2 types) , and CD8^+^ T cells in mice CD45^+^ immune cells. (K) While CRC patients receiving Cetu and grouped with high or low GDH1, IF assay with antibodies against CD11b and Ly6G was performed to verify TANs in human CRC patients’ primary tumors. n=6 clinical CRC samples in each group. (L) Upon Cetu or not, the overall survival of mice injected liver adapted MC38 cells with or without GDH1 was analyzed by a Kaplan‒Meier plot. n=20 mice in each group.

Experiments were repeated at least three times independently with similar results; (N.S., not significant; *p < 0.05; **p < 0.01).

**Supplementary Figure 2.** (A) pGDH1-Y451 (pY451) modification was verified by LC-MS. (B) pY451 peptide and His-tagged GDH1 protein were loaded and blotted with indicated antibody (Upper). GDH1-FLAG (WT or Y451F) in MC38 cells were enriched for WB using indicated antibodies (Lower). (C) The DNA sequence identification of GDH1-Y451 mutating to F451 by CRISPR/Cas9 method. (D) GDH1 depleted HCT116 cells expressing reintroduced GDH1-WT or -Y451F mutant were treated with CHX (2 μM) for indicated time points and then collected for performing WB. The protein decay assay was performed and the data was calculated and analyzed. n=3. (E-F) IF and cytosol-mitochondrial fractionation were performed using GDH1 knockdown HCT116 cells expressing reintroduced GDH1-WT or -Y451F mutant. GDH1 localization was indicated by a GDH1 antibody and MitoTracker (E). Scale bars: 50 μm. The fractionation efficiency and GDH1 location were determined by a WB analysis using antibodies against VDAC1, Tubulin and GDH1 (F). (G) SNU1460 or SNU479 cells were treated with Cetu for 1 or 3 days, then collected for WB using antibodies against ERK1/2 or AKT1 phosphorylation. (H) SNU1460 or SNU479 cells expressing GDH1-WT or -Y451F were treated with or without Cetu for the indicated time points. Cell viability was assessed by trypan blue. n=3.

Experiments were repeated at least three times independently with similar results; (***p < 0.001).

**Supplementary Figure 3.** (A-B) WB (A) and qRT-PCR (B) were performed to confirm the GDH1 expression in GDH1 depleted MC38 or HCT116 cells stably expressing restored GDH1-WT or -Y451E mutant. n=3 biologically independent experiments. (C) GDH1 depleted MC38 cells expressing reintroduced GDH1-WT- or -Y451E were treated with NH_4_Cl (1 μM) for 12 hrs. Co-IP was performed using indicated antibodies. (D) RESPA integrator with 2 fs, 2 fs, and 6 fs timesteps was used for bonding, near, and far. The default value of 9Å was set for the Coulombic cutoff radius. The simulation was run for 100 ns, with a recording interval of 100 ps for energy and trajectory and 1000 frames saved structures. (E) HCT116 cells expressing reintroduced WT- or Y451E-GDH1 were transfected with siHIP1R for 56 hrs. WB was performed using indicated antibodies. (F) MC38 cells were transient with siRNA targeting HIP1R (siHIP1R) or non-target control (siNC), then cytosol and mitochondrial were fractioned for WB using antibodies against GDH1. (N.S., not significant). Experiments were repeated at least three times independently with similar results; data from one representative experiment are shown (A, C-E). (N.S., not significant).

**Supplementary Figure 4.** (A) GDH1-FLAG were enriched for activity determination in GDH1 depleted MC38 or HCT116 cells with restored GDH1-WT-FLAG or -Y451F-FLAG. n=3 biologically independent experiments. (B) KEGG analysis of RNA-sequencing data from restored Y451F-GDH1 versus WT-GDH1 in HCT116 cells. (C) WB tested the OGDH tagged by FLAG and nuclei-located signal (OFN). (D) IF tested the OFN distribution in HCT116 cells. Scale bars: 50 μm. (E) HCT116 cells expressing GDH1-WT or -Y451F were transfected with FLAG/nuclear location signal-tagged OGDH (named OFN) for 48 hrs, then the cytosol, nuclei and mitochondria were sequentially fractioned. Relative αKG was determined. n=3. (F) GDH1 depleted MC38 or HCT116 cells with restored GDH1-WT or -Y451F were transfected with or without OFN for 48 hrs and WB analysis indicated protein expression. (G) ELISA was performed to test CXCL3 level secreted in medium in the above cells in (D). n=3 biologically independent experiments. (H) MC38 or HCT116 cells expressing GDH1-WT or -Y451F were transfected with OFN for 48 hrs or treated with R162 for 12 hrs. Next, the cells were continually treated with Cetu for measuring cell viability. n=3. (I) MC38 or HCT116 cells expressing GDH1-WT or -Y451F mutant were treated with Metformin (1 μM) or Rotenone (50 nM) for 12 hrs, then collected for ATP determination. n=3. (J) Violin plots comparing expression of tumor-secreted Cxcl3 between conditions in tumor cells. (K) Schematic and PCR validation of Cre-mediated recombination at the *Cxcr2* locus. Primers (P1-P3) differentiate intact (P1-P2) and recombined alleles (P1-P3) following tamoxifen induction. β-actin serves as loading control. (L) Western blot analysis confirming specific depletion of *Cxcr2* in neutrophils from *Cxcr2^fl/fl;Ly6g-CreERT2^* mice after tamoxifen treatment. Cre recombinase expression confirms effective genetic modification. β-actin as loading control.

Experiments were repeated at least three times independently with similar results; (*p < 0.05; **p < 0.01; ***p < 0.001).

**Supplementary Figure 5.** (A) In HCT116 cells expressing GDH1-WT or -Y451F, mRNA decay assay was performed by measuring *NUDFA2*, *CXCL3* and *SOS1* mRNA levels using qRT-PCR in the indicated time points. n=3. (B) In HCT116 cells expressing GDH1-WT or -Y451F, RIP-qPCR validated whether ALKBH5 protein binds to *NUDFA2*, *CXCL3* and *SOS1* mRNA. Relative enrichment of *N NUDFA2*, *CXCL3* and *SOS1UDFA2* and *CXCL3* mRNA in each group was shown with the normalization to input. n=3. (C) ELISA was performed to test CXCL3 level secreted in medium while qRT-PCR tested *NUDFA2* and *SOS1* mRNA abundance in HCT116 cells expressing GDH1-WT or -Y451F with or without ALKBH5. n=3. (D) In MC38 cells expressing GDH1-WT or -Y451F mutant, siRNAs targeting FTO or ALKBH5 were transiently transfected for 56 hrs, and collected for WB using antibodies against NUDFA2 and SOS1. (E-F) ATP production (E) and KRAS activity(F) were tested in MC38 or HCT116 cells with or without ALKBH5. (G) RAF1 RBD (Ras-Binding Domain) pulldown assay was performed using MC38 cells with or without indicated adenosine mutation. WB was used to test the efficiency of RAF1 binding KRAS. (H) cell viability was assessed using MC38 or HCT116 cells with or without ALKBH5. n=3. (I) Datamining in TCGA-COAD and READ database was performed to show the mRNA correlation between *ALKBH5* and *NUDFA2*, *CXCL3* or *SOS1* mRNA.

Data present means ± SEM and the statistical analysis was calculated by two-way ANOVA with Tukey’s HSD post hoc test (A and H) and one-way ANOVA with Tukey’s HSD post hoc test (B-C and E-F). (N.S., not significant;). (**p < 0.01; ***p < 0.001).

**Supplementary Figure 6.** (A) The treatment strategy using H-R162 (30 mg/kg) or L-R162 (low-dose of R162, 5 mg/kg) was executed. The xenograft tumors formed by MC38 cells with the indicated treatment or control were recorded and analyzed. n=5 mice in each group. (B) After the execution of R162, the liver function of experimental mice receiving R162 treatment was measured by testing ALT and AST activity. n=5 mice in each group. (C-D) MC38 cells were inoculated into randomized C57BL/6 mice for 1 week, then treated with or without cetuximab alone or with L-R162. The tumor growth was assessed (C). The tumors were collected to fraction the cytosol and nuclei to measure the overall αKG concentration (D). n=5 mice in each group. (E-F) The xenograft tumors (E) or tumors formed by MC38 cells in Figure 6B (F) were collected to extract total proteins and WB tested the indicated protein levels. (G) The tumors formed by MC38 in Figure 6 were shown and the tumor volumes were calculated. (H) The number of tumor associated macrophages was ascertained by anti-CD206 FCM in liver tumors. (I-L) R162 improved the treatment of the combination of cetuximab and KRAS inhibitor. The body weight of mice receiving the combination of indicated treatment (I). Metastatic liver tumors were separated and ATP production(J), CXCL3 secretion (K) and KRAS activity (L) were determined. n=5 mice in each group. Experiments were repeated at least three times independently with similar results; data from one representative experiment are shown (E-F). Data present means ± SEM and the statistical analysis was calculated by two-way ANOVA with Tukey’s HSD post hoc test (A, C) and one-way ANOVA with Tukey’s HSD post hoc test (B, D, F-L). (N.S., not significant;). (*p < 0.05; **p < 0.01; ***p < 0.001).

**Supplementary Figure 7.** (A)The antibody specificity in tissue level was tested by incubating peptide or protein to block the antigen. Scale bars: 200 μm. (B-C) Clinical significance of HIP1R in CRC progression. 49 human CRC samples were collected, and IHC analysis of CRC clinical samples was performed using antibodies against HIP1R. The median positive numbers were set as the cut offs to divide the patients. Representative images of high and low expression of HIP1R were shown in (B, Left), and the IHC score was calculated and analyzed as shown in (B, Right). The overall survival of the CRC patients indicated as HIP1R (C) was analyzed by a Kaplan‒Meier plot. Scale bars: 200 μm. Data present means ± SEM and the statistical analysis was calculated by two-tailed student’s t-test (B) or log rank test (C). (***p < 0.001).

**Supplemental Materials and Methods**

**Plasmids, Reagents and Antibodies**

The plasmids, including pGIPZ-shGDH1#1 and #2 and rescued GDH1, lentivirus packaging and infection, immunoprecipitation (IP) of FLAG-tagged GDH1 for the LC‒MS/MS analysis, GDH1 enzymatic assays were performed as previously described^1,2^. pGEX4T1 and pCDH-3’HA were used as the backbone for GST tagged EGFR (672-1186 amino acids), HIP1R or HA tagged EGFR, respectively while ALKBH5 was cloned into pET28a for generating His-ALKBH5. Constructions were performed using a ClonExpress® Ultra One Step Cloning Kit according to the manual (C115, Vazyme, Nanjing, China). GDH1 Y451F mutation was generated using the QuikChange site-directed mutagenesis kit (Stratagene, La Jolla, CA, USA). shRNA constructs were generated using a pGIPZ backbone digested by XhoI and EcoRI and target sequences were inserted using the T4 DNA Ligase (M0202V, NEB). All the PCR primers and target sequences, the information of reagents and antibodies are shown in the Supplementary Table 2.

**Cell lines**

The cell lines HEK293T and MC38 were purchased from ATCC. The HCT116 cells were kindly provided by Dr. Weiwei Yang (Centre for Excellence in Molecular Cell Science, Chinese Academy of Sciences).

The HEK293T, MC38 and HCT116 cells were cultured in Dulbecco’s modified Eagle’s medium (Invitrogen) containing 10% (v/v) fetal bovine serum (Invitrogen), 1% streptomycin and penicillin. All cells were maintained at 37°C in the presence of 5% CO_2_ in a humidified incubator. No methods were used for cell line authentication.

**Cell viability assay**

In total, 2×10^5^ MC38 or HCT116 cells with or without genetic modifications were plated in DMEM or cetuximab, R162 treatment medium (1 or 10 μM in medium) for the indicated time. Cell viability was determined by Trypan blue staining.

**qRT-PCR**

Quantitative real-time (qRT) PCR analysis was performed using 2×SYBR real-time PCR Premixture (Roche) under the following conditions: 5 mins at 95°C followed by 40 cycles at 95°C for 30 s, 55°C for 40 s, and 72°C for 1 min using an ABI Prism 7700 sequence detection system. The data were normalized to the expression of a control gene (*ACTB*) for each experiment. qPCR primers are shown in the Resources Table.

**Mass spectrometry analysis**

Immunoprecipitated GDH1-FLAG from HCT116 cells with or without half hour cetuximab treatment, or GDH1-FLAG (including WT and Y451E mutant) from HCT116 cells with NH_4_Cl treatment was boiled with 40 μl of 1×loading buffer in 95°C for 8 mins and then loaded into SDS-PAGE gel. The gel samples were processed through series of routine flow, such as reductive alkylation, trypsin digestion and peptide extraction. The peptides were analyzed by liquid chromatography mass spectrometry (LC-MS/MS) on a Q Exactive™ mass spectrometer (Thermo Fisher Scientific, Waltham, MA). Proteins were identified by a database search of the fragment spectra against the National SwissProt protein database (EBI) using Mascot Server 2.4 (Matrix Science, London, UK). After database searching, the intensity of each identified protein was used for further analysis. The data were normalized by log2 foldchange and p-value were calculated between with and without cetuximab treatment groups using two-tailed Student t-test. Tyrosine phosphopeptide matches were analyzed by using MaxQuant v1.5.2.8 implemented in Proteome Discoverer and manually curated. The heatmap plot was constructed using the “pheatmap” package in R 4.2.1 and the colour bar represents the normalized expression level of each site.

**Structure analysis using Desmond**

The structures of human glutamate dehydrogenase 1 (PDB ID: 1L1F, only keep the A chain) were submitted to 100 ns of molecular dynamics simulations using Desmond-v5.7 (D. E. Shaw Research, New York). The crystal structure 1L1F (unphosphorylated and phosphorylated Tyr451) underwent default Protein Preparation Wizard preparation. The minimized structures were solvated to a cubic box with a minimum distance of 10 Å from the protein to the edges. The explicit solvent was described with the TIP3P water model. Systems were neutralized with sodium ions to achieve the final salt concentration of 0.15 M. MD simulations were run with Desmond default settings in the NPT ensemble. The temperature of 300 K was used (Nosé-Hoover chain method). The pressure of 1.01325 bar was maintained with the Martyna-Tobias-Klein method. RESPA integrator with 2 fs, 2 fs, and 6 fs timesteps was used for bonding, near, and far. The default value of 9Å was set for the Coulombic cutoff radius. The simulation was run for 100 ns, with a recording interval of 100 ps for energy and trajectory and 1000 frames saved structures.

**RNA sequencing and analysis**

The total RNA was extracted for RNA sequencing using an Illumina HiSeq 2500 sequencing instrument. The sequencing data analysis and management were performed using the BaseSpace Sequence Hub. A list of differentially expressed genes (DEGs) was obtained according to a p value<0.05 and fold change>1.5 or <0.65.

**Immunohistochemistry (IHC) analysis**

Tissue sections from paraffin-embedded human CRC specimens were stained with antibodies as indicated, and we quantitatively scored the tissue sections according to the percentage of positive cells and staining intensity as previously described^1^. The scores were compared with overall survival, which was defined as the time from the date of diagnosis to death or the last known date of follow-up.

**Immunofluorescence staining and analysis**

CRC and metastatic liver tumour tissues were fixed in 4% formaldehyde and embedded in optimum cutting temperature (OCT) compound (Thermo) after dehydration by 30% sucrose solution. The tissue sections were washed with PBS and 0.3% Triton X-100 and blocked in PBS with 10% normal goat serum, followed by incubation with the indicated antibodies. After washing, the samples were incubated with Alexa Fluor dye-conjugated secondary antibodies and DAPI. The imaging was performed under an Olympus FV3000 confocal laser scanning microscope (Olympus).

Cells were fixed and incubated with the GDH1 antibody, Alexa Fluor dye-conjugated secondary antibodies and DAPI according to standard protocols. The intracellular distribution zone and fluorescence intensity of GDH1 were processed and quantified using ImageJ software ^3,4^, and all data were processed based on original grayscale images. The GDH1 or mitochondrial area was determined by the corresponding dye signals using ImageJ as previously described^5^. Then, the distribution of GDH1 in the mitochondria or cytoplasm was determined by overlapping analysis. The circled area of interest with 32 pixels was selected and measured by ImageJ. The total intensities of 32 pixels were used for further analysis.

**Statistical analysis**

All experiments were repeated independently with similar results from three times and presented as mean ± standard error of the mean (SEM) unless otherwise specified. Statistical analyses were performed using two tailed student’s t-test for pairwise comparison, one-way or two-way analysis of variance (ANOVA) with Tukey’s HSD post hoc test for multiple comparisons and Mann-Whitney U-test for comparison of clinical patients on GraphPad Prism 9. For animal related experiments, mice were randomly assigned to different groups and the assays were results of one-time experiment with statistically sufficient numbers in each group. No statistical method was used to pre-determine the sample size. No data were excluded from the analyses. n values indicated biologically independent experiments or samples. p < 0.05 was considered statistically significant. In all figures, N.S., not significant; *p < 0.05; **p < 0.01; ***p < 0.001.

| **Supplementary Table 1. Clinical and Pathologic Characteristics of CRC Patient** | |
| --- | --- |
| **Variables** | **All patients** |
| **Numbers** | 49 |
| **Gender** |  |
| Male n (%) | 21 (42.8) |
| Female n (%) | 28 (57.2) |
| **Age at diagnosis, yr** | 61.9 ± 10.7 |
| **Year of surgery** | 2016/1-2017/11 |
| **Preoperative CEA, ng/ml** | 0-965 (mean 57.2) |
| **Location** |  |
| Colon n (%) | 32 (65.3) |
| Rectum n (%) | 17 (34.7) |
| **Histology** |  |
| Adenocarcinoma n (%) | 46 (93.9) |
| MAC/SRCC n (%) | 3 (6.1) |
| **AJCC stage** |  |
| II n (%) | 2 (4.1) |
| III n (%) | 26 (53.1) |
| IV n (%) | 21 (42.8) |
| **Grade** |  |
| G1-2 n (%) | 5 (10.2) |
| G3 n (%) | 43 (87.8) |
| Unknown | 1 (2) |
| **Liver Metastasis** |  |
| （-） | 18 (58.1) |
| （+） | 31 (41.9) |
| **Venous invation** |  |
| （-） | 11 (22.4) |
| （+） | 36 (72.6) |
| Unknown | 2 (5) |
| **Perineural invasion** |  |
| （-） | 31 (41.9) |
| （+） | 17 (56.1) |
| Unknown | 1 (2) |
| **Follow-up, months** | 60 |

**Supplementary Table 2. Resources Table**

| **REAGENT OR RESOURCE** | **SOURCE** | **IDENTIFIER** |
| --- | --- | --- |
| Reagents |  |  |
| αKG assay kit | Abcam | ab83431 |
| ATP determination kit | Invitrogen | A22066 |
| MitoTracker Red CMXRos | Invitrogen | M7512 |
| CXCL3 ELISA kit | Abcam | ab234574 |
| Trypan blue solution | Sigma‒Aldrich | T8154 |
| R162 – Calbiochem | Sigma‒Aldrich | 538098 |
| Dimethyl 2-oxoglutarate | Sigma‒Aldrich | 349631 |
| Cetuximab | Selleck | A2000 |
| CQ | Sigma‒Aldrich | C6628 |
| MG-132 | Sigma‒Aldrich | M7449 |
| ALKBH5 | ACTIVEMOTIF (Shanghai, China) | 31589 |
| Lipofectamine 2000 Reagent | Invitrogen | 11668019 |
| RIPA lysis buffer | Millipore | 20-188 |
| Puromycin | MCE | 58-58-2 |
| BAY 87-2243 | TOCRIS | 6980 |
| Rotenone | TOCRIS | 3616 |
| Hygromycin B | MCE | 31282-04-9 |
| GG(m6a) CU were synthesized by Genscript (Nanjing, China) | | |
|  |  |  |
| Antibodies |  |  |
| Mouse monoclonal anti-HA | Sigma-Aldrich | H3663 |
| Mouse monoclonal anti-Flag | Sigma-Aldrich | F4049 |
| Mouse monoclonal anti-Flag M2 affinity gel | Sigma-Aldrich | F1804 |
| Mouse monoclonal anti-β-actin | Sigma-Aldrich | A5316 |
| Mouse monoclonal anti-Tubulin | Sigma-Aldrich | T5168 |
| Rabbit polyclonal anti-ALKBH5 | Sigma-Aldrich | HPA007196 |
| Rabbit anti-m6a antibody | Abcam | ab208577 |
| Rabbit monoclonal anti-FTO | Sigma-Aldrich | ZRB1052 |
| Rabbit polyclonal anti-EGFR | Invitrogen | MA5-13269 |
| Rabbit polyclonal anti-GDH1 | Proteintech | 14299-1-AP |
| Mouse monoclonal anti-GDH1/2 | Santacruz | sc-515542 |
| Rabbit polyclonal anti-VDAC1 | Invitrogen | PA1-954A |
| Rabbit polyclonal anti-Lamin B | Invitrogen | PA5-19468 |
| Rabbit polyclonal anti-RNF213 | ThermoFisher | A305-841A-M |
| Rabbit polyclonal anti-HIP1R | Invitrogen | PA5-58260 |
| Rabbit polyclonal anti-NUDFA2 | Invitrogen | PA5-50462 |
| Rabbit polyclonal anti-CXCL3 | Sigma-Aldrich | AV07037 |
| Rat monoclonal antibody (BM8) to F4/80 | Invitrogen | 13-4801-82 |
| Rat monoclonal [RB6-8C5] to Ly6 g + Ly6c | Abcam | ab25377 |
| PE anti-mouse Arginase 1 Antibody | Biolegend | 165804 |
| Anti-Mo CD206 (MMR) | Invitrogen | 25-2061-82 |
| Rat monoclonal antibody (M1/70) to CD11b | Invitrogen | 12-0112-82 |
| Goat anti-mouse IgG HRP | Invitrogen | G-21040 |
| Goat anti-rabbit IgG HRP | Invitrogen | G-21234 |
| Goat anti-rabbit Alexa Fluor 568 | Abcam | ab175473 |
| Goat anti-mouse Alexa Fluor 555 | Abcam | ab150118 |
|  |  |  |
|  |  |  |
|  |  |  |
| siRNAs | SOURCE | IDENTIFIER |
| Human HIP1R | Thermo Fisher | 216288 |
| Human ALKBH5 | Thermo Fisher | 140422 |
| Human FTO | Thermo Fisher | 272125 |
| Mouse HIP1R | Thermo Fisher | 186405 |
| Mouse ALKBH5 | Thermo Fisher | 153482 |
| Mouse FTO | Thermo Fisher | 166879 |
|  |  |  |
| qPCR primers for human genes | sequence (5' -> 3') |  |
| *GDH1*-forward | GCAGACCCTGCAGGGAATTCA |  |
| *GDH1*-reverse | GCTTTCCGCCCATTCTTGAGT |  |
| *EGFR*-forward | CCCACTCATGCTCTACAACCC |  |
| *EGFR*-reverse | TCGCACTTCTTACACTTGCGG |  |
| *NDUFA1*-forward | GCGTACATCCACAGGTTCACT |  |
| *NDUFA1*-reverse | GCGCCTATCTCTTTCCATCAGA |  |
| *NDUFA2*-forward | GGGACTTCATTGAGAAACGCT |  |
| *NDUFA2*-reverse | AGCATTCGCGGATTAGGATGG |  |
| *NDUFA4*-forward | ATGCTCCGCCAGATCATCG |  |
| *NDUFA4*-reverse | TGCCAGACGCAAGAGATACAG |  |
| *NDUFB2*-forward | GGAGGCCGCCTTTTCAGAA |  |
| *NDUFB2*-reverse | GGAAGGATCAGGATACGGAAAG |  |
| *NDUFB4*-forward | AAACGAGAGTACCTGCTTCAGT |  |
| *NDUFB4*-reverse | AGTGAGTTTTTAGGAGTGGGTCT |  |
| *NDUFA13*-forward | GGCCCATCGACTACAAACGG |  |
| *NDUFA13*-reverse | CGCTCACGGTTCCACTTCATT |  |
| *NDUFB8*-forward | CCGCCAAGAAGTATAATATGCGT |  |
| *NDUFB8*-reverse | TATCCACACGGTTCCTGTTGT |  |
| *NDUFS7*-forward | CTTCGCAAGGTCTACGACCAG |  |
| *NDUFS7*-reverse | GGAATAGTGGTAGTAGCCTCCTC |  |
| *COX5B*-forward | TGTGAAGAGGACAATACCAGCG |  |
| *COX5B*-reverse | CCAGCTTGTAATGGGCTCCAC |  |
| *COX6A1*-forward | TGCTGAATGTGTACCTGAAGTC |  |
| *COX6A1*-reverse | AACGGCTTGGTCCTGATGC |  |
| *CXCL2*-forward | GCAATCCCCGGCTCCTGCGG |  |
| *CXCL2*-reverse | GGTGAATTCCCTGCAGGGTC |  |
| *CXCL3*-forward | TTCACCTCAAGAACATCCAAAGT |  |
| *CXCL3*-reverse | TTCTTCCCATTCTTGAGTGTGGC |  |
| *CXCL10*-forward | GTGGCATTCAAGGAGTACCTC |  |
| *CXCL10*-reverse | GTGGCATTCAAGGAGTACCTC |  |
| *CXCL11*-forward | GACGCTGTCTTTGCATAGGC |  |
| *CXCL11*-reverse | GGATTTAGGCATCGTTGTCCTTT |  |
| *CCL5*-forward | CCAGCAGTCGTCTTTGTCAC |  |
| *CCL5*-reverse | CTCTGGGTTGGCACACACTT |  |
| *CSF1*-forward | TGGCGAGCAGGAGTATCAC |  |
| *CSF1*-reverse | AGGTCTCCATCTGACTGTCAAT |  |
| *CSF2*-forward | TCCTGAACCTGAGTAGAGACAC |  |
| *CSF2*-reverse | TGCTGCTTGTAGTGGCTGG |  |
| *CSF3*-forward | GCTGCTTGAGCCAACTCCATA |  |
| *CSF3*-reverse | GAACGCGGTACGACACCTC |  |
| *IL6*-forward | ACTCACCTCTTCAGAACGAATTG |  |
| *IL6*-reverse | CCATCTTTGGAAGGTTCAGGTTG |  |
| *CXCL8(IL8)*-forward | GTGTGTAAACATGACTTCCA |  |
| *CXCL8(IL8)*-reverse | GCACTGACATCTAAGTTCTTTA |  |
| *OAT1*-forward | TGTTCGGCTACCTTGCAGAC |  |
| *OAT1*-reverse | CGTGTGTGAATGGGCATCCA |  |
| *OAT3*-forward | ATGGCCCAGTCTATCTTCATGG |  |
| *OAT3*-reverse | GACGGTGCTCAGGGTAATGC |  |
| *ACTB*-forward | GCTGGAGTCGATCAACTCTAGG |  |
| *ACTB*-reverse | CCAATTTCGCTTTGCCTTGTC |  |
| qPCR primers for mouse genes | sequence (5' -> 3') |  |
| *CXCL3*-forward | TGAGACCATCCAGAGCTTGACG |  |
| *CXCL3*-reverse | CCTTGGGGGTTGAGGCAAACTT |  |
| *NDUFA2*-forward | ATTTCATCGTGCAACGGTACG |  |
| *NDUFA2*- reverse | GCTCTGGTTACCTCATCAGCA |  |
| *ACTB*-forward | GGATGCAGAAGGAGATTACTGC |  |
| *ACTB*-forward | CCACCGATCCACACAGAGTA |  |
| hOGDH C-terminal knockin FLAG-NLS | sequence (5' -> 3') |  |
| *sgRNA sequence* | CTTCTCGTAGATGCTGCCTA |  |
| Doner DNA sequence | Acggccttcgacctggacgtcttcaag  aacttctcgGGTGGTTCTGGTGA  CTATAAGGACGATGACGATAA  GGGACCTAAGAAGAAGCGAA  AGGTTTAGtgcttctcaactaaagaa  tagtgcctcagcgctg |  |
| Cell lines |  |  |
| HCT116 | ATCC |  |
| MC38 | ATCC |  |
| HEK293T | ATCC |  |

**Reference**

1	Wang, X. *et al.* alpha-Ketoglutarte-Activated NF-kappaB Signaling Promotes Compensatory Glucose Uptake and Brain Tumor Development. *Mol Cell* **76**, 148-162 e147, doi:10.1016/j.molcel.2019.07.007 (2019).

2	Shao, J. *et al.* Cytosolic GDH1 degradation restricts protein synthesis to sustain tumor cell survival following amino acid deprivation. *EMBO J* **40**, e107480, doi:10.15252/embj.2020107480 (2021).

3	Abràmoff M D, M. P. J., Ram S J. . Image processing with ImageJ. *Biophotonics international* **11**, 36-42 (2004).

4	Schneider, C. A., Rasband, W. S. & Eliceiri, K. W. NIH Image to ImageJ: 25 years of image analysis. *Nat. Methods* **9**, 671-675, doi:10.1038/nmeth.2089 (2012).

5	Hamilton, N. Quantification and its applications in fluorescent microscopy imaging. *Traffic* **10**, 951-961, doi:10.1111/j.1600-0854.2009.00938.x (2009).
