# Supplementary material for: α-ketoglutarate accumulation orchestrates immunosuppressive metabolic remodeling to drive cetuximab resistance in metastatic colorectal cancer
Source: Cell Death Dis. 2026 Jun 30;17(1):810. doi: 10.1038/s41419-026-09001-8 (PMC13586316; doi:10.1038/s41419-026-09001-8)
Supplement: Supplementary file 3 [file 41419_2026_9001_MOESM2_ESM.docx]

| **Supplementary Table 1. Clinical and Pathologic Characteristics of CRC Patient** | |
| --- | --- |
| **Variables** | **All patients** |
| **Numbers** | 49 |
| **Gender** |  |
| Male n (%) | 21 (42.8) |
| Female n (%) | 28 (57.2) |
| **Age at diagnosis, yr** | 61.9 ± 10.7 |
| **Year of surgery** | 2016/1-2017/11 |
| **Preoperative CEA, ng/ml** | 0-965 (mean 57.2) |
| **Location** |  |
| Colon n (%) | 32 (65.3) |
| Rectum n (%) | 17 (34.7) |
| **Histology** |  |
| Adenocarcinoma n (%) | 46 (93.9) |
| MAC/SRCC n (%) | 3 (6.1) |
| **AJCC stage** |  |
| II n (%) | 2 (4.1) |
| III n (%) | 26 (53.1) |
| IV n (%) | 21 (42.8) |
| **Grade** |  |
| G1-2 n (%) | 5 (10.2) |
| G3 n (%) | 43 (87.8) |
| Unknown | 1 (2) |
| **Liver Metastasis** |  |
| （-） | 18 (58.1) |
| （+） | 31 (41.9) |
| **Venous invation** |  |
| （-） | 11 (22.4) |
| （+） | 36 (72.6) |
| Unknown | 2 (5) |
| **Perineural invasion** |  |
| （-） | 31 (41.9) |
| （+） | 17 (56.1) |
| Unknown | 1 (2) |
| **Follow-up, months** | 60 |

**Supplementary Table 2. Resources Table**

| **REAGENT OR RESOURCE** | **SOURCE** | **IDENTIFIER** |
| --- | --- | --- |
| Reagents |  |  |
| αKG assay kit | Abcam | ab83431 |
| ATP determination kit | Invitrogen | A22066 |
| MitoTracker Red CMXRos | Invitrogen | M7512 |
| CXCL3 ELISA kit | Abcam | ab234574 |
| Trypan blue solution | Sigma‒Aldrich | T8154 |
| R162 – Calbiochem | Sigma‒Aldrich | 538098 |
| Dimethyl 2-oxoglutarate | Sigma‒Aldrich | 349631 |
| Cetuximab | Selleck | A2000 |
| CQ | Sigma‒Aldrich | C6628 |
| MG-132 | Sigma‒Aldrich | M7449 |
| ALKBH5 | ACTIVEMOTIF (Shanghai, China) | 31589 |
| Lipofectamine 2000 Reagent | Invitrogen | 11668019 |
| RIPA lysis buffer | Millipore | 20-188 |
| Puromycin | MCE | 58-58-2 |
| BAY 87-2243 | TOCRIS | 6980 |
| Rotenone | TOCRIS | 3616 |
| Hygromycin B | MCE | 31282-04-9 |
| GG(m6a) CU were synthesized by Genscript (Nanjing, China) | | |
|  |  |  |
| Antibodies |  |  |
| Mouse monoclonal anti-HA | Sigma-Aldrich | H3663 |
| Mouse monoclonal anti-Flag | Sigma-Aldrich | F4049 |
| Mouse monoclonal anti-Flag M2 affinity gel | Sigma-Aldrich | F1804 |
| Mouse monoclonal anti-β-actin | Sigma-Aldrich | A5316 |
| Mouse monoclonal anti-Tubulin | Sigma-Aldrich | T5168 |
| Rabbit polyclonal anti-ALKBH5 | Sigma-Aldrich | HPA007196 |
| Rabbit anti-m6a antibody | Abcam | ab208577 |
| Rabbit monoclonal anti-FTO | Sigma-Aldrich | ZRB1052 |
| Rabbit polyclonal anti-EGFR | Invitrogen | MA5-13269 |
| Rabbit polyclonal anti-GDH1 | Proteintech | 14299-1-AP |
| Mouse monoclonal anti-GDH1/2 | Santacruz | sc-515542 |
| Rabbit polyclonal anti-VDAC1 | Invitrogen | PA1-954A |
| Rabbit polyclonal anti-Lamin B | Invitrogen | PA5-19468 |
| Rabbit polyclonal anti-RNF213 | ThermoFisher | A305-841A-M |
| Rabbit polyclonal anti-HIP1R | Invitrogen | PA5-58260 |
| Rabbit polyclonal anti-NUDFA2 | Invitrogen | PA5-50462 |
| Rabbit polyclonal anti-CXCL3 | Sigma-Aldrich | AV07037 |
| Rat monoclonal antibody (BM8) to F4/80 | Invitrogen | 13-4801-82 |
| Rat monoclonal [RB6-8C5] to Ly6 g + Ly6c | Abcam | ab25377 |
| PE anti-mouse Arginase 1 Antibody | Biolegend | 165804 |
| Anti-Mo CD206 (MMR) | Invitrogen | 25-2061-82 |
| Rat monoclonal antibody (M1/70) to CD11b | Invitrogen | 12-0112-82 |
| Goat anti-mouse IgG HRP | Invitrogen | G-21040 |
| Goat anti-rabbit IgG HRP | Invitrogen | G-21234 |
| Goat anti-rabbit Alexa Fluor 568 | Abcam | ab175473 |
| Goat anti-mouse Alexa Fluor 555 | Abcam | ab150118 |
|  |  |  |
| siRNAs | SOURCE | IDENTIFIER |
| Human HIP1R | Thermo Fisher | 216288 |
| Human ALKBH5 | Thermo Fisher | 140422 |
| Human FTO | Thermo Fisher | 272125 |
| Mouse HIP1R | Thermo Fisher | 186405 |
| Mouse ALKBH5 | Thermo Fisher | 153482 |
| Mouse FTO | Thermo Fisher | 166879 |
|  |  |  |
| qPCR primers for human genes | sequence (5' -> 3') |  |
| *GDH1*-forward | GCAGACCCTGCAGGGAATTCA |  |
| *GDH1*-reverse | GCTTTCCGCCCATTCTTGAGT |  |
| *EGFR*-forward | CCCACTCATGCTCTACAACCC |  |
| *EGFR*-reverse | TCGCACTTCTTACACTTGCGG |  |
| *NDUFA1*-forward | GCGTACATCCACAGGTTCACT |  |
| *NDUFA1*-reverse | GCGCCTATCTCTTTCCATCAGA |  |
| *NDUFA2*-forward | GGGACTTCATTGAGAAACGCT |  |
| *NDUFA2*-reverse | AGCATTCGCGGATTAGGATGG |  |
| *NDUFA4*-forward | ATGCTCCGCCAGATCATCG |  |
| *NDUFA4*-reverse | TGCCAGACGCAAGAGATACAG |  |
| *NDUFB2*-forward | GGAGGCCGCCTTTTCAGAA |  |
| *NDUFB2*-reverse | GGAAGGATCAGGATACGGAAAG |  |
| *NDUFB4*-forward | AAACGAGAGTACCTGCTTCAGT |  |
| *NDUFB4*-reverse | AGTGAGTTTTTAGGAGTGGGTCT |  |
| *NDUFA13*-forward | GGCCCATCGACTACAAACGG |  |
| *NDUFA13*-reverse | CGCTCACGGTTCCACTTCATT |  |
| *NDUFB8*-forward | CCGCCAAGAAGTATAATATGCGT |  |
| *NDUFB8*-reverse | TATCCACACGGTTCCTGTTGT |  |
| *NDUFS7*-forward | CTTCGCAAGGTCTACGACCAG |  |
| *NDUFS7*-reverse | GGAATAGTGGTAGTAGCCTCCTC |  |
| *COX5B*-forward | TGTGAAGAGGACAATACCAGCG |  |
| *COX5B*-reverse | CCAGCTTGTAATGGGCTCCAC |  |
| *COX6A1*-forward | TGCTGAATGTGTACCTGAAGTC |  |
| *COX6A1*-reverse | AACGGCTTGGTCCTGATGC |  |
| *CXCL2*-forward | GCAATCCCCGGCTCCTGCGG |  |
| *CXCL2*-reverse | GGTGAATTCCCTGCAGGGTC |  |
| *CXCL3*-forward | TTCACCTCAAGAACATCCAAAGT |  |
| *CXCL3*-reverse | TTCTTCCCATTCTTGAGTGTGGC |  |
| *CXCL10*-forward | GTGGCATTCAAGGAGTACCTC |  |
| *CXCL10*-reverse | GTGGCATTCAAGGAGTACCTC |  |
| *CXCL11*-forward | GACGCTGTCTTTGCATAGGC |  |
| *CXCL11*-reverse | GGATTTAGGCATCGTTGTCCTTT |  |
| *CCL5*-forward | CCAGCAGTCGTCTTTGTCAC |  |
| *CCL5*-reverse | CTCTGGGTTGGCACACACTT |  |
| *CSF1*-forward | TGGCGAGCAGGAGTATCAC |  |
| *CSF1*-reverse | AGGTCTCCATCTGACTGTCAAT |  |
| *CSF2*-forward | TCCTGAACCTGAGTAGAGACAC |  |
| *CSF2*-reverse | TGCTGCTTGTAGTGGCTGG |  |
| *CSF3*-forward | GCTGCTTGAGCCAACTCCATA |  |
| *CSF3*-reverse | GAACGCGGTACGACACCTC |  |
| *IL6*-forward | ACTCACCTCTTCAGAACGAATTG |  |
| *IL6*-reverse | CCATCTTTGGAAGGTTCAGGTTG |  |
| *CXCL8(IL8)*-forward | GTGTGTAAACATGACTTCCA |  |
| *CXCL8(IL8)*-reverse | GCACTGACATCTAAGTTCTTTA |  |
| *OAT1*-forward | TGTTCGGCTACCTTGCAGAC |  |
| *OAT1*-reverse | CGTGTGTGAATGGGCATCCA |  |
| *OAT3*-forward | ATGGCCCAGTCTATCTTCATGG |  |
| *OAT3*-reverse | GACGGTGCTCAGGGTAATGC |  |
| *ACTB*-forward | GCTGGAGTCGATCAACTCTAGG |  |
| *ACTB*-reverse | CCAATTTCGCTTTGCCTTGTC |  |
| qPCR primers for mouse genes | sequence (5' -> 3') |  |
| *CXCL3*-forward | TGAGACCATCCAGAGCTTGACG |  |
| *CXCL3*-reverse | CCTTGGGGGTTGAGGCAAACTT |  |
| *NDUFA2*-forward | ATTTCATCGTGCAACGGTACG |  |
| *NDUFA2*- reverse | GCTCTGGTTACCTCATCAGCA |  |
| *ACTB*-forward | GGATGCAGAAGGAGATTACTGC |  |
| *ACTB*-forward | CCACCGATCCACACAGAGTA |  |
| hOGDH C-terminal knockin FLAG-NLS | sequence (5' -> 3') |  |
| *sgRNA sequence* | CTTCTCGTAGATGCTGCCTA |  |
| Doner DNA sequence | Acggccttcgacctggacgtcttcaag  aacttctcgGGTGGTTCTGGTGA  CTATAAGGACGATGACGATAA  GGGACCTAAGAAGAAGCGAA  AGGTTTAGtgcttctcaactaaagaa  Tagtgcctcagcgctg |  |
| Cell lines |  |  |
| HCT116 | ATCC |  |
| MC38 | ATCC |  |
| HEK293T | ATCC |  |
